# Tailoring Versatile Cathodes and Induced Anodes for Zn–Se Batteries: Anisotropic Orientation of Tin‐Based Materials within Bowl‐In‐Ball Carbon

**DOI:** 10.1002/advs.202403224

**Published:** 2024-05-31

**Authors:** Xiaoyu Wu, Xing Chen, Yatao Yan, Guowang Diao, Hui Yan, Lubin Ni, Yuanzhe Piao, Ming Chen

**Affiliations:** ^1^ School of Chemistry and Chemical Engineering Yangzhou University Yangzhou 225002 P. R. China; ^2^ Institutes of Physical Science and Information Technology Anhui University Hefei 230601 P. R. China; ^3^ Department of Chemistry University of Louisiana at Lafayette Lafayette LA 70504 USA; ^4^ Graduate School of Convergence Science and Technology Seoul National University 145 Gwanggyo‐ro, Yeongtong‐gu Suwon‐si Gyeonggi‐do 16229 Republic of Korea; ^5^ Advanced Institutes of Convergence Technology 145 Gwanggyo‐ro, Yeongtong‐gu Suwon‐si Gyeonggi‐do 16229 Republic of Korea

**Keywords:** bidirectional optimization, bowl‐in‐ball carbon structure, multifunctional Se host material, triple induced Zn deposition, Zn–Se batteries

## Abstract

The advancement of Zn–Se batteries has been hindered by significant challenges, such as the sluggish kinetics of Se cathodes, limited Se loading, and uncontrollable formation of Zn dendrites. In this study, a bidirectional optimization strategy is devised for both cathode and anode to bolster the performance of Zn–Se batteries. A novel bowl‐in‐ball structured carbon (BIBCs) material is synthesized to serve as a nanoreactor, in which tin‐based materials are grown and derived in situ to construct cathodes and anodes. Within the cathode, the multifunctional host material (SnSe@BIBCs) exhibits large adsorption capacity for selenium, and demonstrates supreme catalytic properties and spatially confined characteristics toward the selenium reduction reaction (SeRR). On the anode, Sn@BIBCs displays triple‐induced properties, including the zincophilic of the internal metallic Sn, the homogenized spatial electric field from the 3D spatial structure, and the curvature effect of the bowl‐shaped carbon. Collectively, these factors induce preferential nucleation of Zn, ensuring its uniform deposition. As a result, the integrated Zn–Se battery system achieves a remarkable specific capacity of up to 603 mAh g^−1^ and an impressive energy density of 581 W kg^−1^, highlighting its tremendous potential for practical applications.

## Graphical abstract

Carbon in a bowl‐in‐ball structure with internally derived Sn‐based materials is employed for the cathode and anode of Zn–Se batteries, respectively. The SnSe@BIBCs cathode exhibits adsorption, catalytic, and confining properties, while the Sn@BIBCs anode‐induced properties manipulate the uniform deposition of Zn. The simultaneous action of both components contributes to the stability and superior performance of all‐Zn–Se cells.

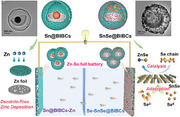

## 1 Introduction

The escalating demand for electrical energy storage, particularly in advanced batteries with significant potential for grid‐scale applications, is driven by the increased utilization of renewable energy sources such as wind and solar power.^[^ ^1^, ^2^, ^3^ ^]^ Currently, the widespread adoption of rechargeable lithium‐ion batteries in the market and industry has been hampered by issues including safety concerns, toxicity risks, and limited resources.^[^ ^4^, ^5^, ^6^ ^]^ As a result, aqueous zinc ion batteries (AZIBs) have recently gained considerable attention as a promising solution for large‐scale storage due to their safety features and the abundance of zinc resources.^[^ ^7^, ^8^, ^9^, ^10^ ^]^ Unfortunately, conventional insert‐type cathode materials, such as vanadium‐based, manganese‐based materials, and Prussian blue analogs, face limitations in achieving the specific capacity required for applications.^[^ ^11^, ^12^, ^13^ ^]^ Moreover, the development of zinc‐ion batteries presents challenges related to dendrite formation, hydrogen evolution issues, and the corrosive effects on the metal anodes.^[^ ^14^, ^15^, ^16^, ^17^ ^]^ These challenges must be overcome to unlock the full potential of zinc‐ion batteries for grid‐scale energy storage.

In accordance with a redox‐based zinc ion storage mechanism, conversion‐type cathodes employing sulfur, selenium, and oxygen/air possess the potential to deliver two to five times larger the capacity than that of cathodes made of ion‐insertion‐type materials.^[^ ^18^, ^19^, ^20^ ^]^ Nevertheless, zinc‐sulfur batteries suffer from a relatively low output voltage, typically falling below 0.6 V.^[^ ^21^, ^22^ ^]^ This voltage limitation represents a significant obstacle to the widespread industrialization of these battery systems. Zinc‐oxygen/air battery systems exhibit subpar cycle performance and currently lack an effective strategy for improvement.^[^ ^23^, ^24^, ^25^ ^]^ Selenium (Se) is a promising candidate as a potential cathode material for rechargeable batteries based on the 6‐electron conversion reaction of Se^4+^/Se^2−^, which has been widely considered and can be applied to AZIBs.^[^ ^26^, ^27^ ^]^ For the conversion‐type cathode, increasing the load of Se often leads to an increased capacity.^[^ ^28^ ^]^ Nevertheless, this approach presents its own set of challenges, including diminished cyclic stability and sluggish electrochemical kinetics due to the substantially large loads. To address these issues and boost capacity while enhancing the dynamics of Se electrodes, researchers have implemented a multifaceted approach, which involves incorporation of layered hollow structures during electrode fabrication, optimizing the specific surface area to mitigate accumulation issues, and integrating highly efficient catalytic materials to lower the energy barriers associated with electrochemical reactions.^[^ ^29^, ^30^ ^]^

The problem in an anode of Zn–Se battery system arises from the growth of zinc dendrites and unfavorable side effects.^[^ ^31^, ^32^, ^33^ ^]^ The uneven distribution of local current densities during zinc deposition has been a major factor contributing to the proliferation of zinc dendrites, significantly impairing Coulombic efficiency (CE) and the overall cycle performance. Moreover, the occurrence of the hydrogen evolution reaction (HER) and the associated volume changes further affect the morphology of the sediment on the zinc metal anode (ZMA), resulting in bubbles or even comminution.^[^ ^34^, ^35^, ^36^ ^]^ To effectively address these challenges, new strategic approaches have been introduced, including rational 3D structure design and advanced manufacturing techniques, as well as the utilization of zincophilic substrates to induce controlled zinc deposition.^[^ ^37^, ^38^, ^39^, ^40^ ^]^

Here, we have fabricated a hollow bowl‐in‐ball carbon (BIBC) structure incorporated with tin‐based materials. This innovative approach includes a bidirectional optimization, enhancing the performance of both cathode and anode in the zinc‐selenium (Zn–Se) battery system. In the cathode, SnSe nanosheets are uniformly dispersed within the hollow spheres and bowls, serving as efficient sites for loading Se that not only facilitate dispersion but also increase the loading capacity. Density functional theory (DFT) simulations have revealed that SnSe exhibits a higher binding energy in comparison to other 2D materials, resulting in efficient adsorption of Se/ZnSe, thus reducing the overall energy barrier for reaction. On the anode, this combination of Sn and BIBC capitalizes the zincophilic properties of tin nanospheres, the homogenized electric field distribution in the hollow structure, and the curvature effect of the bowl‐like structure to facilitate homogeneous zinc deposition.^[^ ^41^, ^42^ ^]^ This meticulous structural design and the tailored cathode/anode incorporated with Sn‐based materials significantly enhance the performance of zinc‐selenium batteries. Furthermore, these findings offer valuable insights that can contribute to the progress of conversion‐type electrodes and the overall improvement of aqueous batteries.

## 2 Results and Discussion

### 2.1 Preparation Process

**Scheme 1:**
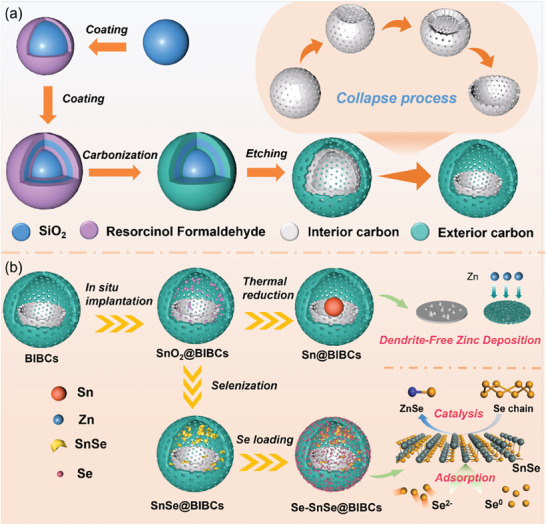
Preparation process of a) BIBCs, b) Sn@BIBCs, SnSe@BIBCs, and Se‐SnSe@BIBCs.

The assembly process of bowl‐in‐ball structure carbon (BIBCs) is depicted in **Scheme** **1**a. Initially, a template consists of SiO_2_ nanospheres synthsized through the hydrolysis of ethyl orthosilicate was employed. Subsequently, a polymerization process involving resorcinol and formaldehyde (abbreviated as RF) was carried out on the SiO_2_ spheres to form a homogeneous coating on their surface. The aforementioned procedure was iteratively executed to yield the SiO_2_@RF@SiO_2_@RF composite material. The precursors were subjected to high‐temperature carbonization within an argon atmosphere to obtain SiO_2_@C@SiO_2_@C composite. After the simultaneous removal of the two SiO_2_ templates, the Double‐Wall Hollow Carbon Spheres (DWHCSs) have been formed. Intriguingly, because the thickness of the inner carbon layer is very thin, the inner carbon sphere is not strong enough to support the spherical structure, inducing its collapse to form a bowl‐like structure. The Bowl‐In‐Ball Carbon (BIBCs) with hollow structure and large specific surface area can be used as an ingenious nanoreactor and serves as an excellent carrier material.

Scheme 1b illustrates the synthetic procedure of tin‐based materials utilizing BIBCs as nanoreactors. First, SnO_2_ nanoparticles were implanted into the BIBCs cavity by in situ confined growth strategy to obtain SnO_2_@BIBCs. Then, two directions of evolution, starting from SnO_2_@BIBCs, can lead to target products with their respective properties. Zncophilic metals, such as Sn, are effective in enhancing uniform zinc deposition, thus, mitigating dendrite growth and extending the operational lifespan. Here, the synthesis of Sn@BIBCs has been achieved via thermal reduction of the precursor (SnO_2_@BIBCs), based on the zincophilic properties of Sn. The second direction is inspired by the fact that carbon material with superior electrical conductivity and 2D metal material with large specific surface area are considered to be suitable host material for selenium. In this process, the SnSe nanosheets in BIBCs are obtained by high‐temperature selenization of the precursor SnO_2_@BIBCs. When acting as a host for Se, the SnSe@BIBCs can simultaneously exert strong adsorption and efficient catalytic properties, which then promote the reaction kinetics and reduce the side reactions.

### 2.2 Design and Characterization

**Figure 1:**
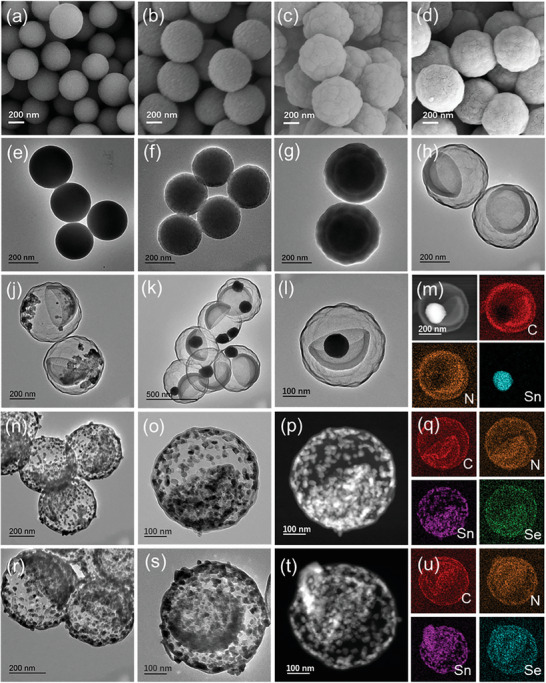
FESEM and TEM images of a,e) SiO_2_ nanospheres, b,f) SiO_2_@RF core‐shell nanospheres, c,g) two‐layer SiO_2_@RF nanospheres, and d,h) BIBCs. TEM images of j) SnO_2_@BIBCs, k,l) Sn@BIBCs. m) HAADF‐STEM images and EDX mapping images of Sn@BIBCs. TEM, HAADF‐STEM, and EDX mapping images of n–q) SnSe@BIBCs and r–u) Se‐SnSe@BIBCs.

The as‐synthesized SiO_2_ nanospheres had a smooth surface and uniform size with a diameter of ≈240 nm, as determined by transmission electron microscopy (TEM) and field emission scanning electron microscopy (FESEM) measurements (**Figure** **1**a,e). With SiO_2_ sphere serving as the inner core, in situ wrappings of RF, SiO_2_, and RF coatings have been conducted sequentially, where the thickness of the two RF coatings comprising 10 nm (Figure 1b,f) and 20 nm (Figure 1c,g), respectively. Ultimately, after high‐temperature carbonization and the removal of silica templates, a delicate small bowl is formed in a hollow carbon sphere (BIBCs), in which the diameter of external hollow spherical shell is ≈350 nm and the depth of the internal bowl‐shaped carbon is ≈130 nm (Figure 1d,h). To demonstrate that it is indeed the thinner internal carbon wall responsible for the formation of bowl structure, a thicker inner wall has been prepared, resulting in a double‐walled hollow sphere structure (Figure S1, Supporting Information). The unique multi‐shell structure of the bowl in hollow ball (BIBC) and double‐wall hollow ball (DWHCS) have considerable large specific surface areas of 1015.4 and 789.2 m^2^ g^−1^, respectively (Figure S2, Supporting Information). The intrinsically porous structure enables easy penetration and transport of guest ions/molecules, while the high specific surface area ensures the binding of electroactive sites for effective electrochemical reaction.

The tiny SnO_2_ nanoparticles developed inside the constrained space of the ball and bowl, partially stacking but not completely filling the void, are demonstrated by TEM and FESEM images (Figure 1j; Figure S3, Supporting Information). The X‐ray diffraction (XRD) pattern, Raman spectra, and thermogravimetric analysis (TGA) curve for SnO_2_@BIBCs are displayed in Figures S4–S6 (Supporting Information). Subsequently, the Sn@BIBCs obtained by thermal reduction have been characterized in detail. TEM images affirm the structural stability of the carbon matrix comprising the bowl‐in‐ball structure after calcination, while metallic tin is fused into nanospheres (Figure 1k,l), attached either to the bowl or the carbon wall. This 3D carbon framework can effectively regulate the local current distribution and the potentially deposited zinc ion flux, and the strong zincophilic inducing effect of Sn nanospheres can facilitate the uniform deposition of Zn. The concave surface of the semi‐open bowl structure induces Zn deposition preferentially because of its curvature effect.^[^ ^42^ ^]^ SEM images corroborate the consistent morphology of Sn@BIBCs and the presence of Sn nanospheres have been observed inside of the sporadic cracks (Figure S7, Supporting Information). High‐resolution transmission electron microscopy (HRTEM) images, XRD pattern, and selected area electron diffraction (SAED) pattern of Sn@BIBCs are explained by the prevalent tetragonal tin phase (Figures S8–S10, Supporting Information). The high angle annular dark field (HAADF)‐STEM image along with elemental mapping data validate the homogeneous distribution of C, N, and Sn elements which are confined in the inner structure (Figure 1m; Figure S11, Supporting Information). The Raman spectrum of Sn@BIBCs is shown in Figure S12 (Supporting Information). Two obvious peaks appear at 1346 cm^−1^ (*I*_D_) and 1594 cm^−1^ (*I*_G_) and the *I*_D_/*I*_G_ ratio is 0.81, indicating that the thermal reduction process further enhances the degree of graphitization of carbon. The X‐ray photoelectron spectroscopy (XPS) spectra in Figure S13 (Supporting Information) demonstrates the chemical composition of the Sn, C, and N elements. Specifically, in the Sn 3d spectrum, two distinct peaks at 485.8 and 494.3 eV correspond to Sn 3d_5/2_ and 3d_3/2_, respectively. The Sn content within the Sn@BIBCs composites has been determined to be 73.8% through the TGA curve, as depicted in Figure S14 (Supporting Information). Likewise, Sn nanospheres exhibit analogous morphologies under the thermal reduction conditions when employing single‐walled and double‐walled carbon spheres, proving the universality of the method (Figures S15 and S16, Supporting Information).

In the other direction, the selenium host materials SnSe@BIBCs have been obtained after high temperature selenization treatment. The scattered SnSe nanosheets are uniformly distributed inside the cavity, enhancing the specific surface area, as depicted in Figure 1n,o. The increase in specific surface area and the presence of a unique multi‐walled carbon can not only enhance the number of loading sites for selenium but also provide a buffer space for selenium expansion and inhibit the escape of polyselenides. HRTEM images, Debye‐Scherrer rings in SAED mode, and characteristic peaks in XRD patterns all attest to the presence of SnSe (Figures S17–S19, Supporting Information). HAADF‐STEM images (Figure 1p) and corresponding mapping results (Figure 1q) exhibit a uniform distribution of C and N elements in the ball and bowl structure. The existence of these aforementioned elements is further confirmed by the EDX pattern of SnSe@BIBCs (Figure S20, Supporting Information). It is pertinent to note that Se is not only evenly distributed in the cavities of both carbon structures and coincident with Sn, but it is also somewhat overlapped with the C element. This is due to the adsorption of selenium vapor on carbon during the high temperature calcination process, and this preloading strategy can further enhance the loading capacity of selenium in the next steps. In addition, we also have synthesized carbon hosts with various morphologies, including dual‐wall hollow spheres, bowl‐shaped carbons, and single‐wall hollow spheres, for comparison. Subsequently, the corresponding SnO_2_@C and SnSe@C complexes have been synthesized and shown in Figures S21–S23 (Supporting Information).

The cathode, made of Se‐SnSe@BIBCs, has been prepared after loading Se into the SnSe@BIBCs host through melt diffusion. When compared to TEM images of the preloaded SnSe@BIBCs, the contrast of SnSe nanosheets and bowl‐in‐ball carbon in Se‐SnSe@BIBCs is deepened after being loaded with Se (Figure 1r,s). The Se element on carbon appears to be amorphous, proving that Se can be embedded in mesoporous pores in the carbon wall, in addition to being loaded on the surface of BIBCs (Figure S24, Supporting Information).^[^ ^43^ ^]^ Elemental analysis reveals the overlap of Se with Sn, C, and N elements, indicating the binding of Se in C and SnSe nanosheets (Figure 1t,u; Figure S25, Supporting Information). Thus, SnSe is capable of adsorbing selenium when employed as a host material. Differences in Raman spectra between Se‐SnSe@BIBCs, SnSe@BIBCs and commercial selenium powders have been observed. The shift of characteristic peak of Se from 237 to 255 cm^−1^ is due to the Se preloading on BIBCs, while the increase of intensity of the characteristic peak in Se‐SnSe@BIBCs further confirms the effective adsorption for Se by the preloading strategy (Figure S26, Supporting Information). XPS spectra, before and after the Se loading, provide additional support (Figures S27 and S28, Supporting Information). In particular, the Se 3d_5/2_ and Se 3d_3/2_ peaks shift from 53.2 and 54.7 eV to 53.9 and 55.2 eV, respectively, which are likely caused by the strong interaction between the host (SnSe) and the guest (Se). More impressively, when compared with Se 3d in SnSe@BIBCs, the ratio of peak areas for Se 3d_5/2_ and Se 3d_3/2_ is significantly increased, indicating an increase of Se° content in the Se‐SnSe@BIBCs (Figures S27c and S28c, Supporting Information). Figure S29 (Supporting Information) demonstrates that even after selenium loading and infiltration, the specific surface area of Se‐SnSe@BIBCs remains high (344.7 m^2^ g^−1^, Table S1, Supporting Information), positively affecting its contact with electrolyte and thus, electron transport between the two. The preloading approach is responsible for the high load capacity of 65.4 wt.% of Se, as determined by comparing the TGA curves of Se before and after loading (Figures S30 and S31, Supporting Information). In addition, Se has been successfully deposited into other carbon substrates of various shapes (BIBCs, DWHCSs, carbon bowl) for comparison, and TEM images demonstrate the homogeneous loading of Se on all structures (Figures S32–S34, Supporting Information).

### 2.3 Coin‐Cell Performances of the Cathode Materials

**Figure 2:**
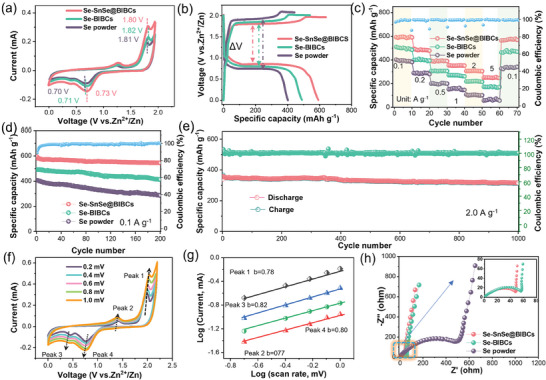
a) Cyclic voltammetry (CV) curves at 0.5 mV s^−1^ b) initial GCD profiles at 0.1 A g^−1^ (voltage window 0–2 V) and c) Rate performance to compare voltage polarization and specific capacity of Se‐SnSe@BIBCs, Se‐BIBCs and Se powder electrodes. d) Cycling performances of three electrodes at 0.1 A g^−1^. e) Cycling performances of Se‐SnSe@BIBCs at 2 A g^−1^. f) CV curves at various scan rates and g) corresponding log *i* versus log *v* plot at redox peak of Se‐SnSe@BIBCs. h) Nyquist plots of three electrodes.

According to the aforementioned characterization, selenium has been effectively contained in SnSe nanosheets and the carbon framework. Se‐SnSe@BIBCs was used as a cathode to assemble into a coin cell for electrochemical performance testing. The two pairs of peaks in the cyclic voltammetry curves correspond to the redox processes of Se^4+^/Se^0^ and Se^0^/Se^2−^, respectively (**Figure** **2**a; Figure S35, Supporting Information). The Se‐SnSe@BIBCs cathode demonstrates the highest reduction and the lowest oxidation peak potentials of 0.73 and 1.80 V, respectively, when compared to Se‐BIBCs (0.71 V, 1.82 V) and Se powder (0.70 V, 1.81 V). The results strongly confirm the occurrence of catalytic reaction. The catalytic ability of SnSe can lower the energy barrier of the conversion reaction of Se, thus, accelerating the kinetics of Se transformation and reducing the degree of electrochemical polarization. Figure 2b shows a consistency between the platforms of the galvanostatic charge and discharge (GCD) of the three materials and the CV curves. In particular, Se‐SnSe@BIBCs present the superior discharge characteristics and capacity, achieving 614.8 mAh g^−1^, when compared to those of Se@BIBCs at 527.9 mAh g^−1^ and Se powder at 406.5 mAh g^−1^.

Upon further assessment of the rate performance, it is evident that across all tested rates (0.1–5 A g^−1^), the Se‐SnSe@BIBCs cathode maintains the highest specific capacity among the three samples, even at a high current density of 5 A g^−1^, with a capacity of 271.6 mAh g^−1^ (Figure 2c). To validate the enhanced stability attributed to the bowl‐in‐ball structure and the catalytic advantage of SnSe, the Se‐SnSe@BIBCs and the other samples underwent performance analysis and were compared after 200 cycles at a low current density of 0.1 A g^−1^. Although the Se powder electrodes display a swift degradation in capacity, both the Se‐SnSe@BIBCs and Se‐BIBCs electrodes maintain remarkable cyclic stability after 200 cycles, by sustaining specific capacities of ≈579.2 and 443.5 mAh g^−1^, respectively (Figure 2d). The reversible capacity of Se‐SnSe@BIBCs is noteworthy, maintaining at 328.1 mAh g^−1^ even after 1000 cycles at a high current density of 2 A g^−1^, thereby, highlighting its outstanding cyclic stability (Figure 2e). Remarkably, even under an exceptionally high current density of 5 A g^−1^, the Se‐SnSe@BIBCs electrode retains a discharge capacity of 256.7 mAh g^−1^ after 800 cycles, signifying an impressive performance with 88.6% capacity retention (Figure S36, Supporting Information). Stable cycling performance highlights the versatility of SnSe@BIBCs as a host, including the adsorption and efficient catalysis of SnSe, and the confined effect of the bowl‐in‐ball structure.

Through the examination of CV curves at different scanning rates, the equations *i* = *av^b^* and log (*i*) = *b*log (*v*) + log (*a*) are employed to examine the behavior of the electrode (Figure 2f). In accordance with the linear correlation observed between the response current and sweep rate, an ideal capacitance process is characterized by a *b* value of 1.0, while the full diffusion control behavior is associated with a *b* value of 0.5. The oxidation and reduction peak in the Se‐SnSe@BIBCs electrode exhibit *b* values notably close to 1, suggesting that the predominant reaction mechanism is the capacitive process (Figure 2g). Moreover, the Nyquist curve derived from electrochemical impedance spectroscopy (EIS), offering insights into charge transfer reactions and diffusion processes at the electrode/electrolyte interface (Figure 2h), which further accentuate the rapid kinetics demonstrated by Se‐SnSe@BIBCs. This heightened kinetic behavior can be attributed to the electrocatalytic properties of SnSe, coupled with the substantial specific surface area inherent in its unique structural configuration.

### 2.4 Theoretical Calculation and Mechanism Study

**Figure 3:**
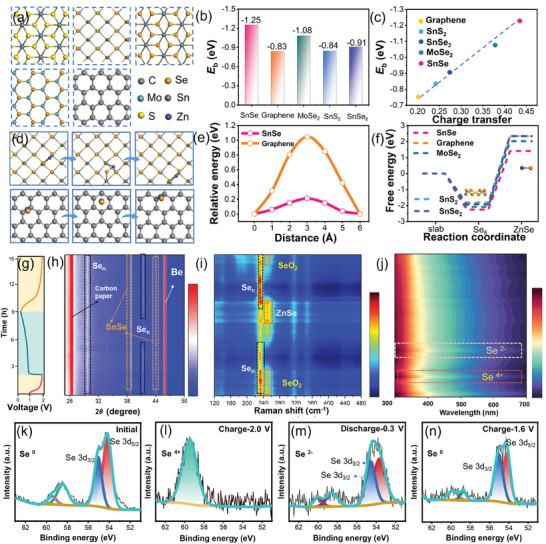
Theoretical calculation and mechanism study. a) Modeling, b) Summary of the calculated *E*_b_, c) The linear relationship between *E*_b_ and charge transfer of Zn atom of graphene, SnS_2_, SnSe_2_, MoSe_2_ and SnSe. d) Schematic diagram of diffusion paths for ZnSe migration e) energy barrier of graphene and SnSe. f) Free energy plots of conversion reactions of isolated ZnSe/Se. g) Galvanostatic charge/discharge (GCD) curve of the Se‐SnSe@BIBCs cathode at 0.2 A g^−1^ and in situ h) XRD patterns, i) Raman, and j) UV–vis spectra for the Zn–Se cells. Ex situ k–n) high‐resolution XPS spectra for Se 3d.

The superior catalytic performance of SnSe is further demonstrated by performing DFT calculations on selected common 2D materials (**Figure** **3**a; Figure S37, Supporting Information). The calculated binding energies (*E*_b_) between zinc atoms and various substances are summarized in Figure 3b. Notably, SnSe displays the strongest binding energy (*E*_b_) among those selected 2D materials. The charge transfer rates of those selected materials are compared in Table S2 (Supporting Information), in which SnSe owes the largest charge transfer rate, illustrating that SnSe can accelerate the reaction kinetics. In addition, a linear relationship between *E*_b_ and charge transfer is observed in Figure 3c. The deposition and decomposition of ZnSe are linked to the utilization rate of selenium cathode and the reversibility of zinc‐selenium battery. To further investigate these processes, we conducted simulations of the diffusions of ZnSe on SnSe and graphite. Figure 3d,e illustrates the diffusion paths and corresponding migration energy barriers for both materials, measured at 0.22 and 1.14 eV, respectively. These findings affirm that the inclusion of SnSe facilitates the transformation of ZnSe. The Gibbs free energy barrier is relevant to the difficulty associated with the ZnSe/Se_8_ transformation reaction. As depicted in Figure 3f, selected 2D materials reduce the free energy of the reaction, and SnSe displays the lowest Gibbs free energy (2.31 eV) among them. This emphasized its catalytic role in facilitating the reaction.

To gain deeper insights into the storage mechanism of Zn^2+^ within the cathode of Se‐SnSe@BIBCs, we have conducted a detailed analysis of the solid‐phase evolution and in‐solution transition of Se during a 0.2 A g^−1^ GCD cycle (Figure 3g), with a series of in situ tests. The complete patterns of in situ XRD between 20° and 50° are presented in Figure 3h. Two peaks at 26.4° and 46.3° persist consistently throughout the process, attributed to current collector and XRD equipment, respectively. During the discharge of the Se‐SnSe@BIBCs electrodes to 0.3 V, the characteristic peak of selenium at 41.7° (100) nearly vanishes, signifying the conversion of Se.^[^ ^27^ ^]^ Subsequently, those peaks gradually reappear during charging, indicating a highly reversible process. Furthermore, no discernible shift is observed in the distinctive peaks of SnSe at 38.8° and 44.1° throughout the redox process, suggesting that SnSe retains catalytic capabilities. Correspondingly, the Raman spectrum shows the characteristic peak of SeO_2_ at 251.1 cm^−1^ during the first charging process.^[^ ^26^ ^]^ As the discharge process proceeds, the SeO_2_ and Se phases disappear in sequence, and the peak appearing at 253.4 cm^−1^ is assigned to ZnSe. In the second charging process, ZnSe gradually diminishes until it completely disappears, while the Se phase is regenerated as seen in the evolution of Raman feature at 234 cm^−1^, and in a similar pattern, SeO_2_ resurfaces as charging continues (Figure 3i; Figure S38, Supporting Information). Meanwhile, in situ Ultraviolet–visible (UV‐Vis) spectroscopy has been employed to investigate the physical phase transition in solution (Figure 3j). It is worth noting that, during the discharge process, no distinct peaks representing other selenides in solution have been observed, except for the presence of trace amount of ZnSe. In the charging process, absorption peaks of Se^4+^ appear, further confirming the presence of SeO_2_. This observation convincingly demonstrates that the catalytic and adsorption properties of SnSe, coupled with the confined effect of the hollow structure, contribute to the high efficiency of Se conversion. Furthermore, it can effectively immobilize and limit the escape of selenides in the electrolyte (Figure S39, Supporting Information).

The redox reactions occurring at the selenium cathode have been further investigated using ex situ XPS. At the first charging at 2.0 V, the Se 3d spectrum shows a sharp peak located at 59.4 eV, which is attributed to the oxidation process of the conversion from Se^0^ to Se^4+^ (Figure 3k,l). Upon discharging the battery at 0.3 V, the peaks at 53.6 (Se 3d_5/2_) and 54.8 eV (Se 3d_3/2_) are assigned to Se^2−^, as demonstrated in Figure 3m. Lastly, the Se 3d spectrum has been selected for charging to 1.6 V, which displays two distinct peaks at 54.1 (Se 3d_5/2_) and 55.2 eV (Se 3d_3/2_), indicating the oxidation of Se^2‐^ to Se^0[^ ^28^ ^]^ (Figure 3n). Hence, the mechanism of redox transformation of zinc and selenium is illustrated by the six‐electron reversible interconversion of Se. The catalytic ability of SnSe and the abundance of sites in the multi‐shell structure becomes crucial to boost the reversibility of the conversion reaction. To substantiate the inhibitory effect of SnSe@BIBC on the adsorption and shuttle effect of polyselenides, the Se‐SnSe@BIBCs electrode was immersed in the electrolyte solution to monitor the color change. In situ UV–vis spectroscopy of the solution shows that no polyselenides has been produced or any by‐products is soluble in the solution (Figure S40, Supporting Information). A series of in situ/ex situ characterization methods, coupled with DFT simulations have provided compelling evidence to support the proficient catalytic role of SnSe in enabling the reversible conversion between Se and ZnSe. The distinctive adsorption characteristics exhibited by SnSe can effectively suppress the dissolution of polyselenides, thereby making a substantial contribution to the enhanced performance of the Zn–Se battery.

### 2.5 Electrochemical Properties of Sn@BIBCs‐Zn Anodes

**Figure 4:**
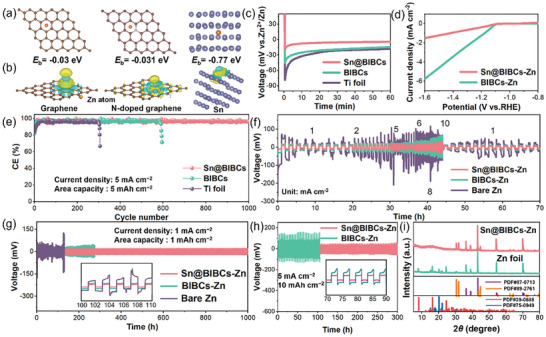
The binding energy (*E*_b_) of the a) geometric structures and b) charge density difference (CDD). c) Voltage profiles of Zn plating on Ti foil, BIBCs‐Zn and Sn@BIBCs‐Zn. d) LSV curves of Sn@BIBCs and BIBCs for HER. e) CE plots of different hosts. GCD voltage profiles of different cells at f) different current densities, g) at 1 mA cm^−2^ for 1 mAh cm^−2^, and h) 5 mA cm^−2^ for 10 mAh cm^−2^. i) XRD patterns of bare Zn and Sn@BIBCs‐Zn before and after cycling in symmetric cells with the aqueous ZnSO_4_ electrolyte (reference data from the corresponding JCPDS files: PDF#87‐0713 for Zn, PDF#89‐2761 for Sn, and PDF#39‐0688 for Zn_4_SO_4_(OH)_6_·4H_2_O).

DFT calculations were performed to acquire an insight into the functional role of the elements within Sn@BIBCs in nucleation and the evolution of zinc deposition. The binding energies (*E*_b_) between zinc atoms and various hosts in the Sn@BIBCs system are illustrated in **Figure** **4**a and Figure S41 (Supporting Information). Particularly, Sn exhibits the strongest binding energy (−0.77 eV) with zinc atoms, surpassing those of graphene and N‐doped graphene, demonstrating its zincophilic property. Furthermore, Sn and Zn atoms demonstrate the most substantial charge distribution and polarization at the interface, as revealed by DFT‐based charge density difference (CDD) calculations (Figure 4b). This strong affinity and efficient electron transfer between Zn and Sn atoms can promote the deposition of zinc on Sn@BIBCs, effectively inhibiting the growth of zinc dendrites.

The results from theoretical calculations underscore the significant potential of Sn@BIBCs in the modification of the property of zinc metal to serve as electrodes. To validate the superior performance of the Sn@BIBCs, a series of electrochemical tests on Zn‐Sn@BIBCs were conducted. Zinc foil and Sn@BIBCs‐Zn have been characterized prior to electrochemical testing (Figures S42–S44, Supporting Information). First, the energy barriers of Zn nucleation on Sn@BIBCs, BIBCs, and Ti foils at a low current density of 1 mA cm^−2^ are shown in Figure 4c. Remarkably, the Sn@BIBCs exhibits a noteworthy small nucleation overpotential of 20.5 mV, which is lower than that of Ti foil (78.3 mV) and BIBCs (49.6 mV), highlighting the superior zincophilic property of Sn@BIBCs. Furthermore, the nucleation overpotentials of carbon and Sn composites with other morphologies are listed in Table S3 (Supporting Information), which demonstrates that the bowl‐in‐ball structure also plays an auxiliary role in reducing the nucleation potential barrier. In addition, Sn@BIBCs displays the lowest stripping overpotential among the tested samples, proving their superiority as zinc metal modification material (Figure S45, Supporting Information).

Linear sweep voltammetry (LSV) distinctly reveals that Sn@BIBCs present lower electrocatalytic activity for the hydrogen evolution reaction (HER) in comparison to BIBCs (Figure 4d). This characteristic is conducive to reducing the occurrence of hydrogen evolution side reactions. The HER activity of bare zinc foil is more pronounced, leading to the generation of H_2_ bubbles in the presence of a ZnSO_4_ solution (Figure S46, Supporting Information). The wettability test of ZnSO_4_ electrolyte can reflect the Zn ion migration ability of Sn@BIBCs. The contact angle of the electrolyte on the Sn@BIBCs surface is 37.1°, as depicted in Figure S47 (Supporting Information), significantly lower than that of the zinc foil (77.5°). This distinct reduction of the contact angle implies the strong capillary action on the surface of Sn@BIBCs, which is beneficial to the transverse growth of Zn crystals. The reversibility of the conversion of these electrodes has been evaluated by assembling Zn‖Ti asymmetric batteries. Figure 4e demonstrates the exceptional cycle stability of Sn@BIBCs, maintaining an average Coulombic efficiency (CE) of 99.7% at 5 mA cm^−2^ and 5 mAh cm^−2^ over 1000 cycles without any noticeable fading. In addition, at 5 mA cm^−2^, the CEs of 1 and 2 mAh cm^−2^ are maintained at 99.4% for 600 cycles and 99.1% for 500 cycles, respectively, far surpassing the performance of BIBCs and Ti foil (Figure S48, Supporting Information).

Long‐term cycling stabilities of symmetric cells using Sn@BIBCs‐Zn, BIBCs‐Zn and bare Zn electrodes were investigated. Compared with electrodes made with BIBCs‐Zn and bare zinc foils, the one with Sn@BIBCs‐Zn exhibits the most superior performance at various current densities ranging from 0.1 to 5 mA cm^−2^ in terms of voltage hysteresis (Figure 4f). The Sn@BIBCs‐Zn electrode operates continuously for 1000 h with a low voltage lag of 33 mV at a current density of 1 mA cm^−2^, as illustrated in Figure 4g. The bare Zn and BIBCs‐Zn electrodes display the significant oscillation of polarization voltage after cycling for ≈124 and 281 h, respectively, which may be attributed to short circuits or detrimental side reactions. Even when subjected to higher area capacities and larger discharge depths (5 mA cm^−2^, 10 mAh cm^−2^, Figure 4h), the symmetric cell employing Sn@BIBCs‐Zn electrode can still run stably at a relatively low overpotential for 150 h, while the voltage polarization of BIBCs‐Zn is more noticeable. The symmetric cells with Sn@BIBCs‐Zn electrodes also depict stable behavior when varying area capacities and discharge depths (Figure S49, Supporting Information), further demonstrating that Sn@BIBCs with ball‐in‐bowl configuration and zincophilic Sn can effectively improve zinc plating/stripping and extend the life of ZMAs.

To evaluate the effect of Sn@BIBCs induction, the stability of zinc metal anodes during deposition and stripping, we have investigated the morphological change and valence state variation after the first deposition/stripping by SEM and XPS. After the first deposition of Zn, SEM images and cross‐sectional SEM images depict a uniform thickness of the deposited layer without any significant bumps (Figures S50–S52, Supporting Information). The XPS spectra of Sn@BIBCs‐Zn further confirm the strong interaction between Sn and deposited zinc, as evident from the noticeable displacements in Sn 3d_5/2_ and 3d_3/2_ peaks after zinc deposition (Figure S53, Supporting Information). More importantly, the morphology, after the first stripping, reveals that zinc can be stripped completely from the electrodes without the presence of obvious “dead” zinc. This relies on the robust 3D structure of Sn@BIBCs and its well‐distributed electric field strength, which promotes excellent reversibility of the deposition/stripping process (Figures S54 and S55, Supporting Information). In contrast, unmodified Zn foil exhibits significant irregularities and dendrite formation after the initial deposition, persisting even after stripping and resulting in an irreversible state (Figures S56–S58, Supporting Information). In addition, in situ optical observations provide more direct evidence. Specifically, during the deposition process, Sn@BIBCs can effectively average the electric field and inhibit the formation of dendrites, with negligible changes on the surface (Figure S59a and Video S1, Supporting Information). On the contrary, the surface of zinc foil changes dramatically under the same conditions. In the initial stage (10 min), some small protrudes appear on the surface of the zinc foil and rapidly evolve into zinc dendrites (Figure S59b and Video S2, Supporting Information). The zincophilic characteristics of Sn, coupled with the bowl structure, allow the deposited zinc particles to be uniformly and flatly distributed on Sn@BIBCs, which provide ideal nucleation sites for subsequent zinc deposition.

Furthermore, the surface morphologies of Sn@BIBCs‐Zn and pure Zn after 20 and 100 h plating/stripping in symmetric cells at various current densities are described by SEM images (Figures S60–S63, Supporting Information). The numerous disordered zinc clusters are shown on the bare zinc anode, attributed to the uneven nucleation on the bare zinc foil. This phenomenon not only results in potential short‐circuiting but also accelerates the HER rate. On the contrary, the surface of Sn@BIBCs‐Zn anode reveals minimal changes and remains stable throughout the cycling period. This is due to the large amount of space within the Sn@BIBCs as well as the semi‐open bowl structure that relieves stress during cycles, thereby making the electrode surface more robust.

To verify their excellent property of corrosion resistance, two electrodes have been immersed in ZnSO_4_ electrolyte for 72 h. Based on XRD patterns, hydrated zinc sulfide by‐products are generated at the bare zinc electrode, whereas no secondary product generation is observed at the Sn@BIBCs‐Zn electrode (Figure 4i). These findings have been further confirmed by optical photographs and SEM images, clearly illustrating the differences between the two materials following immersion (Figures S64 and S65, Supporting Information). Consequently, the Sn@BIBCs can serve not only as a nucleation agent to induce the uniform deposition of Zn but also as a corrosion inhibitor, which can effectively prevent surface corrosion.

To summarize, the excellent performance of Sn@BIBCs‐Zn electrode has been attributed to the following reasons: 1) the zincophilicity of Sn with HER inertia deceases the nucleation overpotential and induces the Zn deposition. 2) Asymmetrical bowl structure induces Zn deposition preferentially due to its curvature effect. 3) the layered 3D conductive network homogenizes the spatial electric field, hence enhancing the induction of Zn. Therefore, the special triple induction mechanism greatly improves homogeneous zinc deposition and enhances cycling stability of Sn@BIBCs‐Zn electrode.

### 2.6 Performance of Zn||Se Full and Pouch Cells

**Figure 5:**
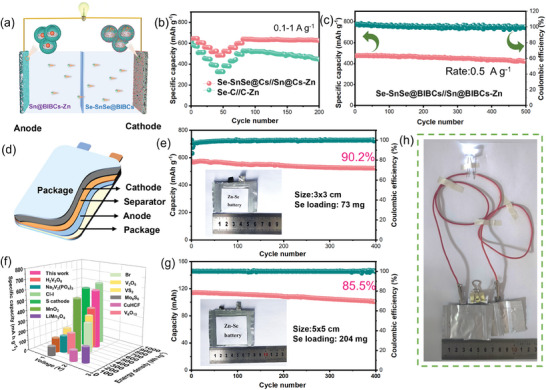
a) Schematic illustration of the Sn@BIBCs‐Zn||Se‐SnSe@BIBCs cell. b) Rate performances, c) Cycling performances of full cell at 0.5 A g^−1^. d) Schematic picture of the Zn–Se pouch cell. Cycling performance of e) 73 mg and g) 204 mg Se loading pouch cell using Se‐SnSe@BIBCs cathode. f) Comparison of voltage, specific capacity, and energy density of different cathode materials for zinc‐ion batteries. h) The white LED (drive potential: 3 V) is powered by pouch cells in series.

The zinc‐selenium battery system has been assembled employing Sn@BIBCs modified Zn foil as the anode and Se‐SnSe@BIBC as the cathode, in accordance with the excellent performance (**Figure** **5**a; Figure S66, Supporting Information). The electrocatalytic effect of the cathode (SnSe) and the uniform homogenous induction effect of the anode (Sn) support are accounted for the exceptional rate performance (Figures 5b). After cycling at various current densities from 0.1–1 A g^−1^, the average capacity of Sn@BIBCs‐Zn||Se‐SnSe@BIBCs is 632.5 mAh g^−1^, substantially surpassing that of C‐Zn||Se‐C (403.4 mAh g^−1^) after 100 cycles when the current density returns to 0.1 A g^−1^. The reversible capacity maintains 436 mAh g^−1^ after 500 cycles at a current density of 0.5 A g^−1^, as illustrated in Figure 5c. In view of the excellent coin full battery performance of Zn–Se, we have extended our efforts to pouch cell systems with various loads and areas, which have been fabricated as depicted in Figure 5d. The prepared 73 mg selenium‐loaded pouch cells were cycled at 0.2 A g^−1^. In 400 cycles, the specific capacity of the cell exceeds 500 mAh g^−1^, the volume retention is 91.1%, and the Coulombic efficiency approaches 100% (Figure 5e). To demonstrate the effectiveness of high selenium loadings, we also fabricated a pouch cell with a cathode area of 25 cm^2^ and a loading of 203 mg. The results show that the Zn||Se pouch cell obtains a high specific energy density of 581 Wh kg^−1^ and a stable cycle life, maintaining 90.6% of the initial capacity in 400 cycles with a Coulombic efficiency close to 100% (Figure 5g). The comparative analysis with previous reports, considering discharge capacity, voltage, and energy density, has demonstrated that our work is clearly superior to many other cathodes made of various materials (Figure 5f).^[^ ^18^, ^44^, ^45^, ^46^, ^47^, ^48^, ^49^, ^50^, ^51^, ^52^ ^]^ Finally, the practical application of zinc‐selenium battery system is illustrated by connecting three pouch cells in series to power up an LED light (Figure 5h). The above results confirm that the synergistic effect of the cathode Se‐SnSe@BIBCs and the anode Sn@BIBCs effectively improve the performance of the full battery, indicating the effectiveness of bidirectional derivative strategies of the tin‐based material in the bowl‐in‐ball structure.

## 3 Conclusion

In this paper, we have developed bidirectional derivative strategies of the tin‐based material in the bowl‐in‐ball structure. Initially, SnO_2_ nanoparticles has been implanted into a unique bowl‐in‐ball hollow carbon structure. The subsequent bidirectional evolution fabricates the multifunctional Se host material of SnSe@BIBCs and an inducible Zn deposition material of Sn@BIBCs, respectively. On one hand, our investigations have substantiated the multiple functions of SnSe@BIBCs, including high‐load Se with preloading strategy, and catalytic property for selenium conversion reaction. Meanwhile, the structural advantage of SnSe@BIBCs imparts a confinement effect, which can prevent the escape of selenium. In situ characterization fully comprehend the reaction mechanism of Zn–Se cells and identify the crucial factors contributing to their exceptional performance. On the other hand, Sn@BIBCs exhibit the unique triple‐induced mechanism for the uniform deposition of Zn, i.e., contained zincophilic‐induced deposition, uniform electric field‐induced deposition, and structure‐induced deposition. The process ensures satisfactory coulomb efficiency, preventing the formation of zinc dendrites or other undesired by‐products. The fully assembled battery system, featuring optimized cathode and anode components, has demonstrated exceptional performance, achieving a capacity of up to 603 mAh g^−1^ and an impressive energy density of 581 Wh kg^−1^. These results underscore the strong potential of our battery system, and serve as a valuable reference for a broader application of tin‐based materials in energy storage, the design of innovative hollow structures, and the advancement of zinc‐selenium battery technology.

## 4 Experimental Section

### Synthesis of BIBCs

To commence the synthesis, a solution was formulated by blending 65 mL of ethanol with 15 mL of deionized water. To this mixture, 3 mL of ammonia, 3 mL of tetraethyl orthosilicate, 0.6 g of resorcinol, and 1.15 mL of formaldehyde were introduced. The resulting solution underwent stirring for 20 h. Subsequently, a new solution was created by combining 6 mL of ammonia, 6 mL of tetraethyl orthosilicate, 1.1 g of resorcinol, 1.52 mL of formaldehyde, and 120 mg of the previously obtained SiO_2_@RF composite. This mixture was added to a solution containing 150 mL of ethanol and 18 mL of deionized water to achieve homogeneity. Magnetic stirring was conducted for 40 h, followed by centrifugation at 6000 rpm for 10 min. This process yielded the composite material SiO_2_@RF@SiO_2_@RF. The composite material underwent calcination in a tube furnace at a temperature of 700 °C for 6 h, followed by cooling to ambient temperature. Subsequently, the silica component was removed using a NaOH solution at 60 °C, resulting in the final product, BIBCs. These carbon bowls were synthesized under identical temperature and etching conditions using SiO_2_@RF as a precursor.

### Synthesis of SnSe@BIBCs,Se‐SnSe@BIBCs and Sn@BIBCs

The synthesis began by dissolving 0.51 g of Na_2_[Sn(OH)_6_] and 1.0 g of urea in a mixture comprising 80 mL of deionized water and 30 mL of ethanol. Subsequently, 75 mg of BIBCs was dispersed into the solution using ultrasonic dispersion for 1 h. The resulting suspension was then transferred into a Teflon autoclave to initiate a solvothermal reaction, maintained at 160 °C for 14 h. Throughout this process, a black product was formed, collected by centrifugation, and subsequently washed with deionized water and ethanol to eliminate impurities. The SnO_2_@BIBCs product was then transferred to a vacuum oven and dried overnight at 60 °C under vacuum conditions. In the final step, SnO_2_@BIBCs were combined with appropriate Se powders and placed in a ceramic vessel for 6 h at 350°C. This heating process took place in a tubular furnace under an Ar/H_2_ atmosphere with a ratio of 95:5. Se‐SnSe@BIBCs was synthesized by sealing SnSe@BIBCs and Se powder in a quartz tube and heating at 260 °C for 12 h. Similarly, Se‐BIBC was prepared by the melt diffusion method described above.

### Material Characterization

Transmission Electron Microscopy (TEM) images were captured using a Philips TECNAI‐12 instrument. Field‐emission Scanning Electron Microscopy (FESEM) images were obtained using a Hitachi S‐4800 instrument from Japan. High‐resolution Transmission Electron Microscopy (HRTEM) and High‐angle Annular Dark‐field Scanning Transmission Electron Microscopy (HAADF‐STEM) were carried out using a FEI Tecnai G2 F30 STWIN instrument operating at 300 kV. X‐ray diffraction (XRD) data were collected using a graphite monochromator and Cu Kα radiation (*λ* = 0.1541 nm) on a D8 advance superspeed powder diffractometer from Bruker. The X‐ray Photoelectron Spectroscopy (XPS) experiments were performed using a Thermo Escalab 250 system with Al Kα radiation (1486.6 eV). N_2_ adsorption and desorption measurements were carried out at 77 K using a volumetric method and a MicroMetrics ASAP 2020 Plus surface area & porosity analyzer. Prior to measurement, samples were degassed at 150 °C for 6 h under vacuum. Specific surface area was determined via the Brunauer–Emmett–Teller (BET) method using adsorption data, and pore size distribution was derived from the adsorption branch using the Barrett–Joyner–Halenda (BJH) method. The UV–vis spectra were recorded on a Shimadzu UVmini‐1280 spectrophotometer. Thermogravimetric Analysis (TGA) measurements were conducted in a Netzsch TG209 F3 instrument over a temperature range of 25 to 900 °C, with a heating rate of 5 °C min^−1^.

### Electrochemical Characterization

Cyclic voltammetry (CV), linear sweep voltammetry (LSV), and electrochemical impedance spectroscopy (EIS) measurements were conducted using an Electrochemistry Workstation (CHI660 E, manufactured by Chenghua, China) with a scanning rate of 0.5 mV s^−1^ and a voltage range of 0.01–2.0 V. Additionally, an Autolab Electrochemical Analyzer (Ecochemie, Netherlands) was employed for these electrochemical tests. Charge–discharge tests were performed utilizing a battery test system (CT‐3008 W, produced by Xinwei, China).

### In Situ Characterization

To perform in situ XRD characterization, a cell module was fitted with a beryllium window. The slurry was applied onto the ultrathin titanium foil, which was securely affixed to the beryllium window. The cathode was composed of a Se‐SnSe@BIBCs electrode arranged parallel to the Zn anode. Both electrodes were separated by a conventional glass fiber separator saturated with electrolyte. The Se‐SnSe@BIBCs cathode was positioned at the top for X‐ray exposure. In situ Raman spectroscopy was carried out using a confocal Raman spectrometer. Batteries, for in situ UV–vis spectrometry, were assembled using a customized in situ colorimetric dish with Se‐SnSe@BIBCS electrode as the cathode and Zn metal as the anode. UV‐vis spectra were recorded every 15 min from the beginning to the end of the discharge. UV–vis absorption spectroscopy (UV–vis, Shimadzu UVmini‐1280 spectrophotometer) is used to detect the concentration of substances and the chemical state of the elements.

### Computational Details

First‐principles calculations were performed using the Vienna Ab‐initio Simulation Package (VASP). The Perdew–Burke–Ernzerhof (PBE) generalized gradient approximation (GGA) was utilized for the exchange‐correlation energy calculation. To account for the van der Waals (vdW) interactions in the systems, the DFT‐D3 method developed by Grimme was applied. The Projector Agmented–Wave (PAW) method with a plane‐wave basis set with an energy cut‐off of 450 eV was used for total energy calculations. The force and energy convergence criterions were set to be 0.02 eV Å^−1^ and 10^−5 ^eV, respectively. To eliminate interactions between adjacent unit cells, a vacuum separation >15 Å was employed. The first Brillouin Zone was sampled using a k‐point mesh of 3 × 3 × 1. The binding energy (*E*_b_) of zinc atoms was defined as: *E*_b_ = *E*_slab+Se_ − *E*_slab_ − *E*_Se_, where *E*_slab_ is the energy of Graphene, SnS_2_, SnSe_2_, MoSe_2_, and SnSe, respectively. *E*_Se_ is the energy of Se, and *E*_slab+Se_ is the total energy of the Se adsorbed on the corresponding surface. The climbing‐image nudged elastic band (CI‐NEB) method was employed to calculate the barriers for ZnSe diffusion on Graphene and SnSe, respectively.

## Conflict of Interest

The authors declare no conflict of interest.

## Supporting information

Supporting Information

Supplemental Video 1

Supplemental Video 2

## Data Availability

The data that support the findings of this study are available from the corresponding author upon reasonable request.
