# Supplementary material for: Tailoring Versatile Cathodes and Induced Anodes for Zn–Se Batteries: Anisotropic Orientation of Tin‐Based Materials within Bowl‐In‐Ball Carbon
Source: Adv Sci (Weinh). 2024 May 31;11(29):2403224. doi: 10.1002/advs.202403224 (PMC11304292; doi:10.1002/advs.202403224)
Supplement: Supplementary file 1 — Supporting Information [file ADVS-11-2403224-s003.docx]

Supporting Information

Tailoring Versatile Cathodes and Induced Anodes for Zn-Se Batteries: Anisotropic Orientation of Tin-based Materials within Bowl-in-ball Carbon

*Xiaoyu Wu^a^,* *Xing Chen^b^, Yatao Yan^a^,* *Guowang Diao^a^*, Hui Yan^c^*, Lubin Ni^a^, Yuanzhe Piao^d,e^*, Ming Chen^a^**

**Content**

1. Supplementary figure (Figure S#).
2. Process diagram of double wall hollow ball structure and bowl in ball structure. TEM images of (a)(b) SiO_2_@RF, (c)(d) SiO_2_@RF@SiO_2_@RF, (e) double-walled hollow carbon sphere, (f) bowl in ball carbon.
3. Nitrogen adsorption-desorption isothermal curves and pore size distribution of the DWHCSs (a, b) and BIBCs (c, d).
4. FESEM images of (a) SnO_2_@BIBCs and SnSe@BIBCs
5. XRD pattern of SnO_2_@BIBCs.
6. Raman spectrum of SnO_2_@BIBCs.
7. TGA curve of the SnO_2_@BIBCs.
8. SEM images of Sn@BIBCs.
9. HRTEM images of Sn@BIBCs.
10. XRD pattern of Sn@BIBCs.
11. SAED pattern of Sn@BIBCs.
12. EDX pattern of Sn@BIBCs.
13. Raman spectrum of Sn@BIBCs.
14. XPS spectra of Sn@BIBCs: (a) survey (b) C 1s (c) Sn 3d
15. TGA curve of the Sn@BIBCs.
16. TEM images of Sn@HCSs.
17. TEM images of Sn@DWHCSs.
18. HRTEM images of SnSe@BIBCs.
19. SAED pattern of SnSe@BIBCs.
20. XRD pattern of SnSe@BIBCs and Se-SnSe@BIBCs.
21. EDX pattern of SnSe@BIBCs.
22. TEM images of (a) SnO_2_@DWHCSs and (b) SnSe@DWHCSs.
23. TEM images of (a) Carbon bowls (CBs) (b) SnSe@CBs.
24. TEM images of hollow carbon spheres (HCSs, (a, b)), SnO_2_@HCSs (c, d), and SnSe@HCSs (c, f).
25. HRTEM images of Se-SnSe@BIBCs
26. EDX pattern of Se-SnSe@BIBCs.
27. Raman spectra of Se-SnSe@BIBCs, Se@BIBCs and Pure Se.
28. XPS spectra of SnSe@BIBCs: (a) survey, (b) C 1s, (c) Se 3d, and (d) Sn 3d.
29. XPS spectra of Se-SnSe@BIBCs: (a) survey, (b) C 1s, (c) Se 3d, and (d) Sn 3d
30. Nitrogen adsorption-desorption isothermal curves and (b) pore size distribution of the SnSe@BIBCs
31. TGA curve of the SnSe@BIBCs.
32. TGA curve of Se-SnSe@BIBCs.
33. TEM images of Se-BIBCs.
34. TEM images of Se-DWHCSs.
35. TEM image of Se-carbon bowl.
36. Comparison of CV curves of Se-SnSe@BIBCs, Se-BIBCs and Se powder electrodes
37. Cycling performances of Se-SnSe@BIBCs at 5 Ag^-1^.
38. Side view of (a) SnS_2_, (b) SnSe, (c) SnSe_2_, (d) MoSe_2_ and (e) graphene
39. Raman spectrum of initial electrolyte, (b) *in situ* Raman spectra at first cycle.
40. UV−vis absorption spectrum of initial electrolyte.
41. *In situ* UV spectra of electrolyte in static state.
42. Side view of Sn and Zn models in DFT calculation.
43. Optical photo images of (a) zinc foil and (b) Sn@BIBCs-Zn.
44. SEM images of (a-b) zinc foil and (c-d) Sn@BIBCs-Zn.
45. Cross-sectional images of Sn@BIBCs-Zn.
46. Voltage profiles of Zn stripping from Ti foil, BIBCs and Sn@BIBCs.
47. (a) LSV curve for HER and (b) photograph of Zn foil in ZnSO_4_.
48. Wettability tests using ZnSO_4_ electrolyte on (a) Sn@BIBCs-Zn and (b) Zn foil
49. CE plots of different electrodes at 5 mA cm^-2^ at a capacity of (a) 1 mAh cm^-2^ and (b) 2 mAh cm^-2^.
50. Cycling performances of symmetric cells at various density.
51. Zn deposition behaviors on the Sn@BIBCs (a) before and (b) after Zn plating for 2 mAh cm^-2^.
52. SEM images of Zn deposition behaviors on the Sn@BIBCs for 5 mAh cm^-2^.
53. Cross-sectional images of Zn deposition behaviors on the Sn@BIBCs for 5 mAh cm^-2^.
54. XPS spectra of Sn@ BIBCs during Zn plating. XPS spectra of (a) Sn 3d before and after Zn plating and (b) Zn 2p after Zn deposition.
55. SEM image of the Sn@BIBCs-Zn after stripping with a capacity of 5 mAh cm^-2^.
56. Cross-sectional images of the Sn@BIBCs-Zn after stripping with a capacity of 5 mAh cm^-2^.
57. Zn deposition behavior on Zn foil. SEM images of the surfaces of Zn foil for 5 mAh cm^-2^.
58. Cross-sectional images of Zn deposition behaviors on Zn foil for 5 mAh cm^-2^Zn deposition behaviors on the Sn@BIBCs.
59. Zn stripping behavior on Zn foil. SEM images of the surface of Zn foil for 5 mAh cm^-2^.
60. In situ optical observations of the Zn plating process on (a) Sn@BIBCs-Zn and (b) Zn foil as a function of time at a current density of 10 mA cm^−2^.
61. SEM images of Sn@BIBCs-Zn plating/stripping at a current density of 1 mA cm^−2^ on 20 h.
62. SEM images of Zn plating/stripping at a current density of 1 mA cm^−2^ on 20 h.
63. SEM images of Sn@BIBCs-Zn plating/stripping at a current density of 5 mA cm^−2^ at 100 h.
64. SEM images of Zn plating/stripping at a current density of 5 mA cm^−2^ at 100 h.
65. Optical photo images of (a) bare Zn foil and (b) Sn@BIBCs-Zn after immersion in the aqueous ZnSO_4_ electrolyte for 72 hours.
66. SEM images of (a) bare Zn foil and (b) Sn@BIBCs-Zn after immersion in the aqueous ZnSO_4_ electrolyte for 72 hours.
67. GCD profiles at 0.1 C of the Sn@BIBCs-Zn||Se-SnSe@BIBCs cell.

1. **Supplementary figure**

**1.1** **Process diagram of double wall hollow ball structure and bowl in ball structure.**


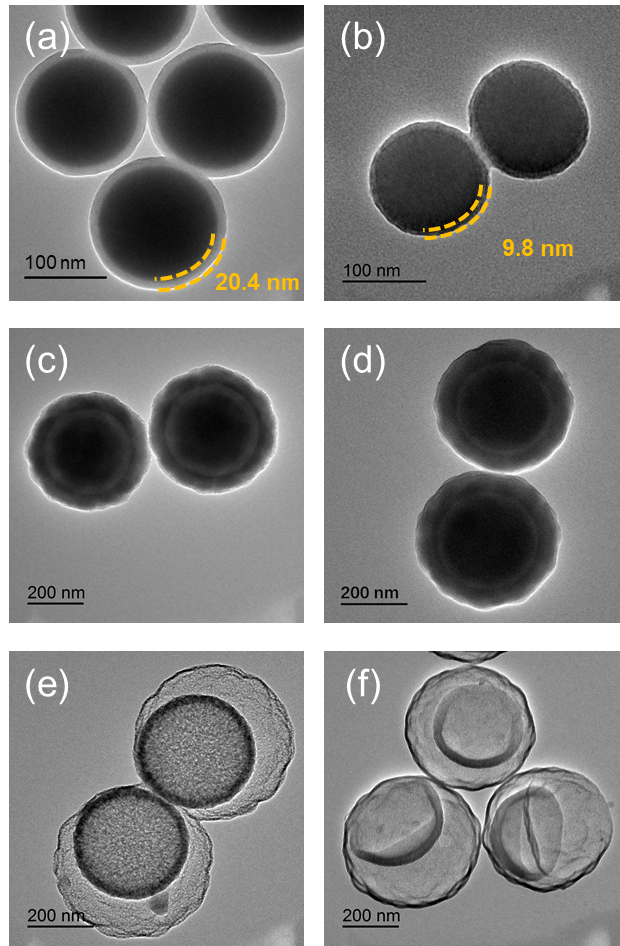


**Figure S1** TEM images of (a)(b) SiO_2_@RF, (c)(d) SiO_2_@RF@SiO_2_@RF, (e) double-walled hollow carbon sphere, (f) bowl in ball carbon.

**
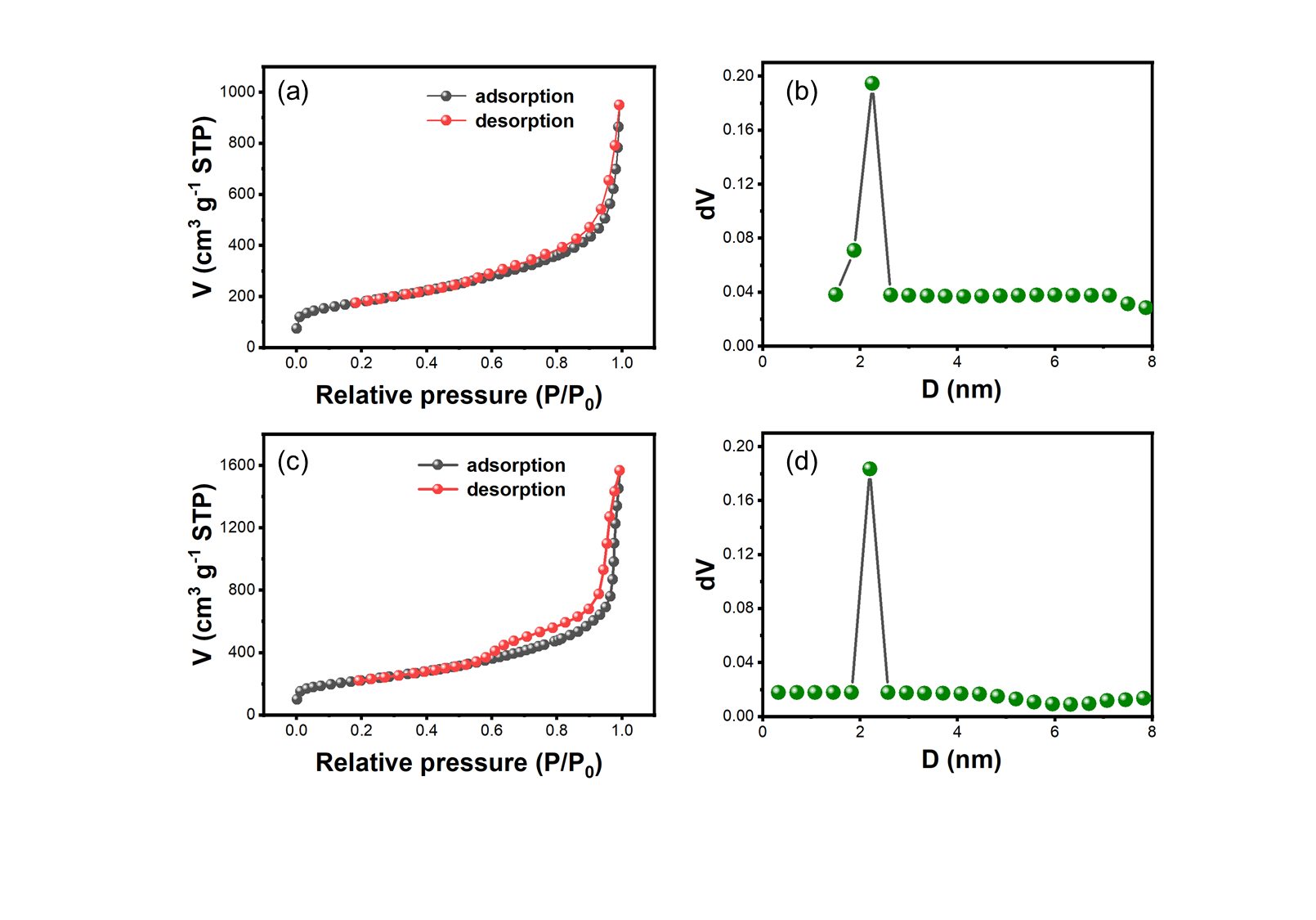
**

**Figure S2** Nitrogen adsorption-desorption isothermal curves and pore size distribution of the (a) (b) DWHCSs and (c) (d) BIBCs.

**Note**: As shown by the N_2_ adsorption/desorption isotherm and pore size distribution, the bowl in hollow ball and double-wall hollow ball materials has mesoporous structures that permit subsequent ions to pass through the carbon shell and deposit into the cavity. The Brunauer-Emmet-Teller surface area of the double-walled hollow sphere is 789.2 m^2^g^−1^, and the specific surface area of the bowl-like structure in the sphere is even as high as 1015.4 m^2^g^−1^, which is due to the mass loss caused by the thinning of the carbon wall.

**1.2 The characterization of SnO_2_@BIBCs**

**
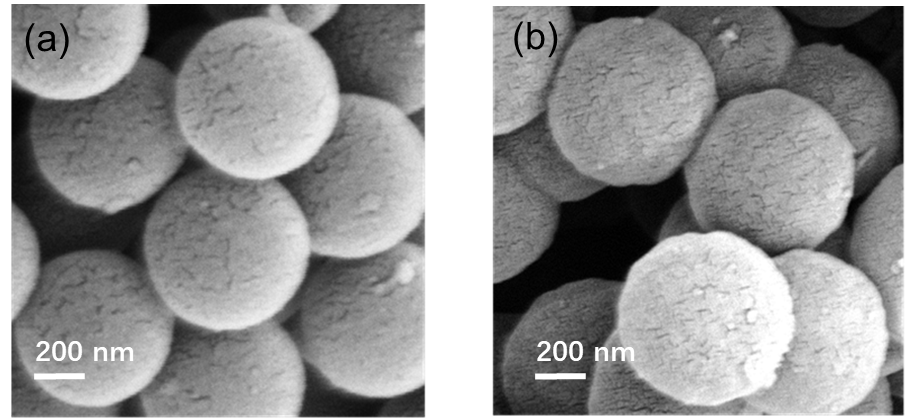
**

**Figure S3** FESEM images of (a) SnO_2_@BIBCs and SnSe@BIBCs.

**
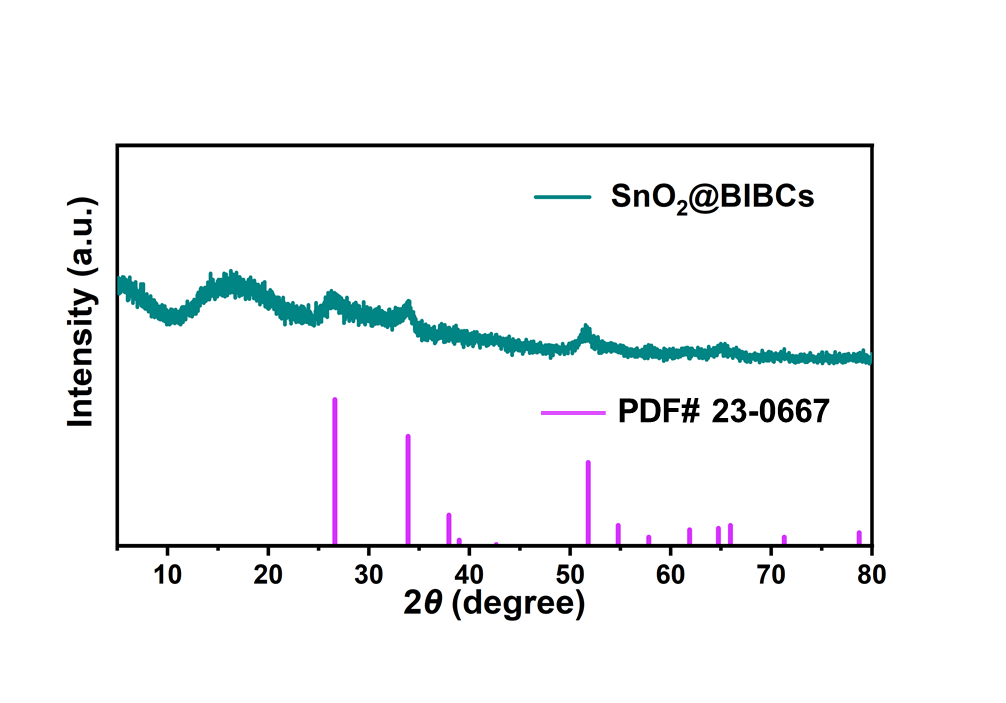
**

**Figure S****4** XRD pattern of SnO_2_@BIBCs

**Note**: The X-ray diffraction (XRD) pattern for SnO_2_@BIBCs is displayed in **Figure S4**, which features notable peaks that are adequately indexed to SnO_2_ (PDF # 23-0667).

**
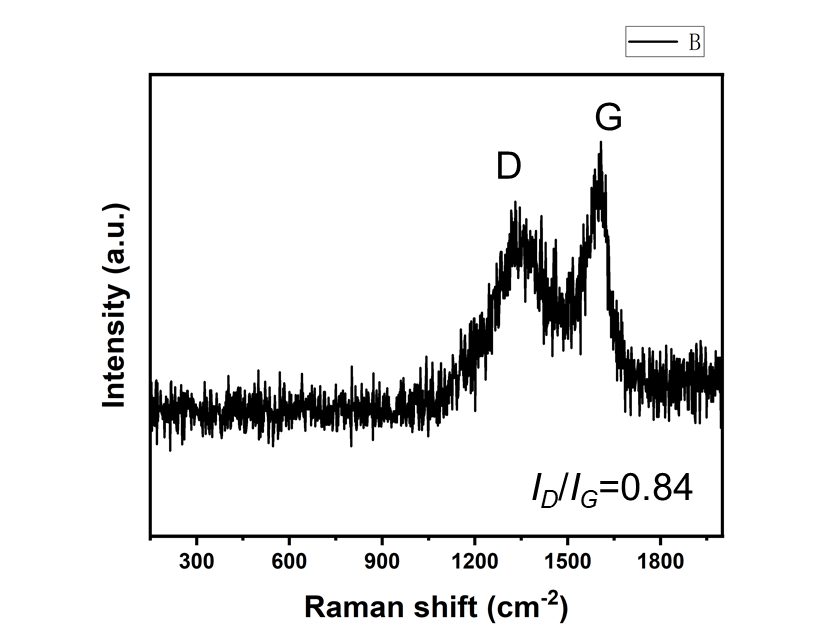
**

**Figure S5** Raman spectrum of SnO_2_@BIBCs

**Note**: Raman spectra reveal *D* and *G* bands at approximately 1345 cm^−1^ and 1590 cm^−1^, respectively, with an *I_D_/I_G_* ratio of 0.84, indicating the presence of defective or disorderly carbon (**Figure S5**).


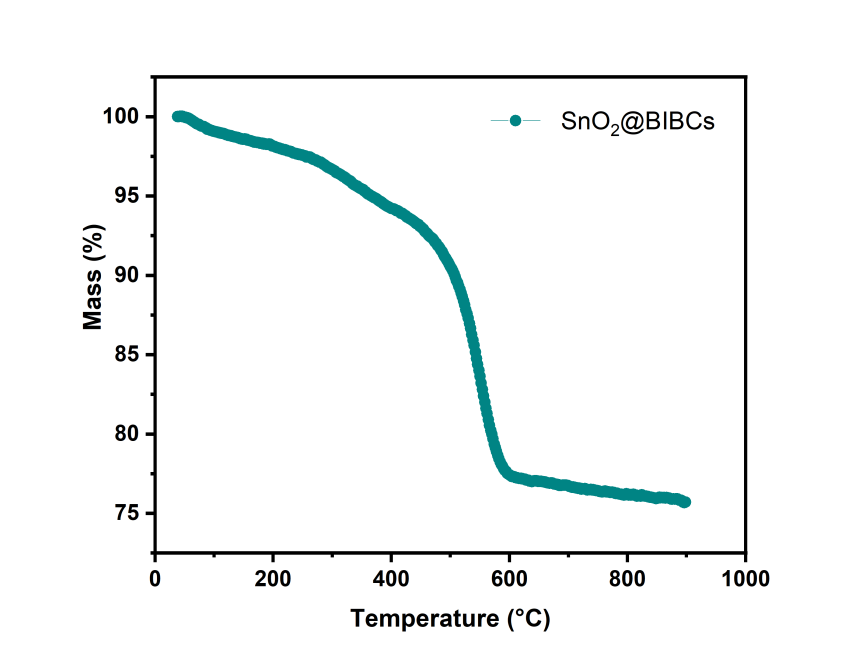


**Figure S6** TGA curve of the SnO_2_@BIBCs. (The weight content of the SnO_2_ is 74.7%)

**Note**: The thermogravimetric analysis (TGA) curve of SnO_2_@BIBCs is shown in **Figure S6**, accounting for approximately 74.7% of the total mass.

**1.3 The characterization of Sn@BIBCs**

**
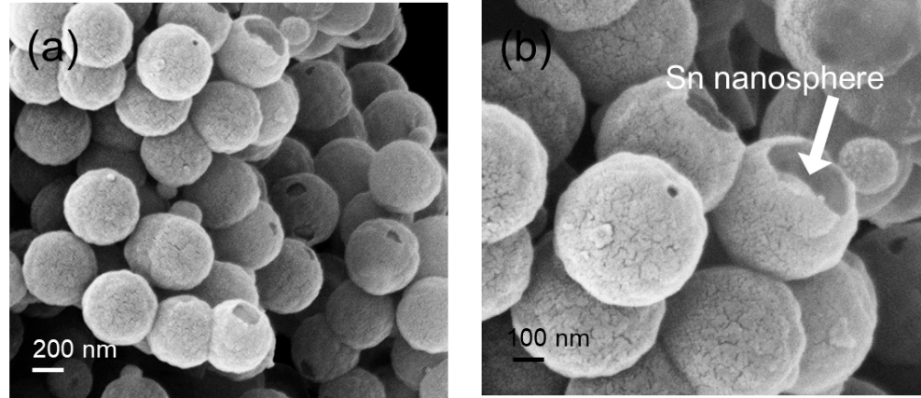
**

**Figure S7** SEM images of Sn@BIBCs.


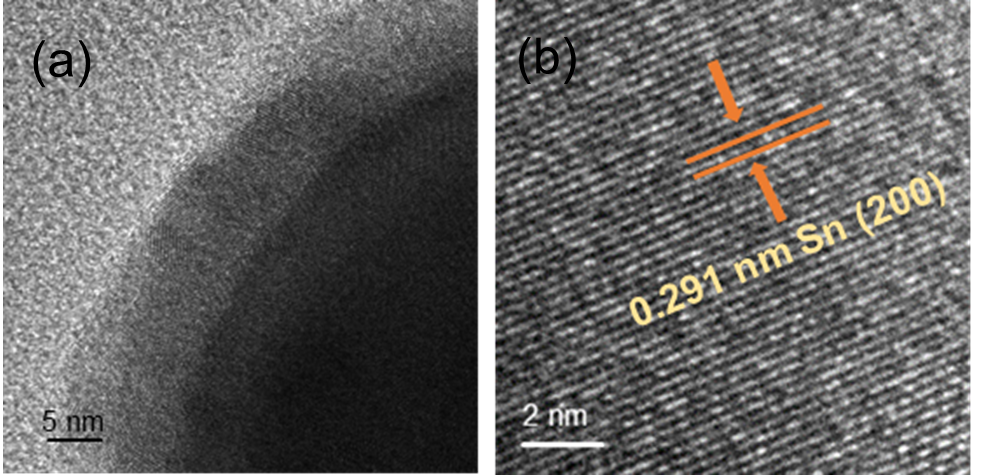


**Figure S8** HRTEM images of Sn@BIBCs

**
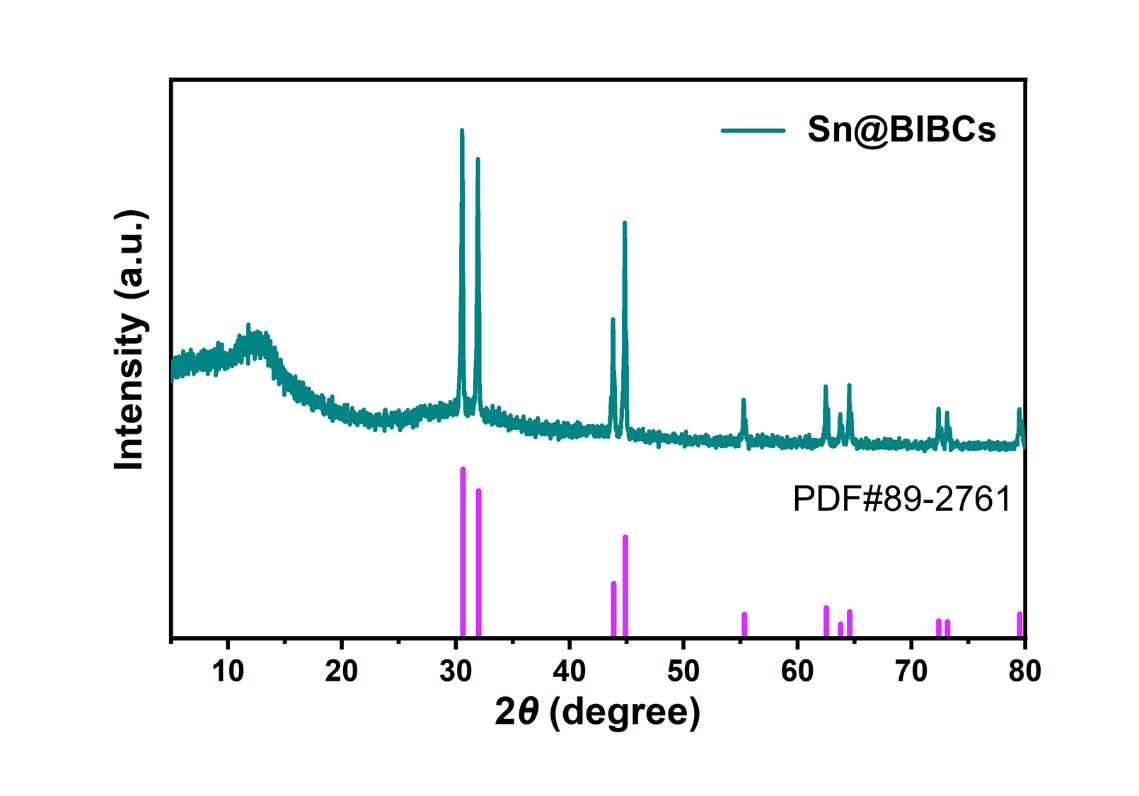
**

**Figure S9** XRD pattern of Sn@BIBCs.

**
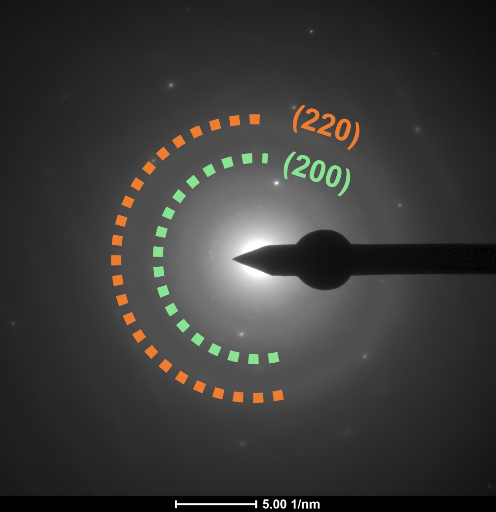
**

**Figure S10** SAED pattern of Sn@BIBCs.

**Note**: **Figures S8** displays HRTEM images revealing *D*-spacing of the (200) lattice fringe, measured at 0.291 nm, corresponding to the distinctive peaks discerned in the XRD pattern (**Figure S9**). Debye-Scherrer ring patterns associated with (220) and (200) are evident in the SAED pattern of Sn@BIBCs, attributed to the prevalent tetragonal tin phase (**Figure S10**).


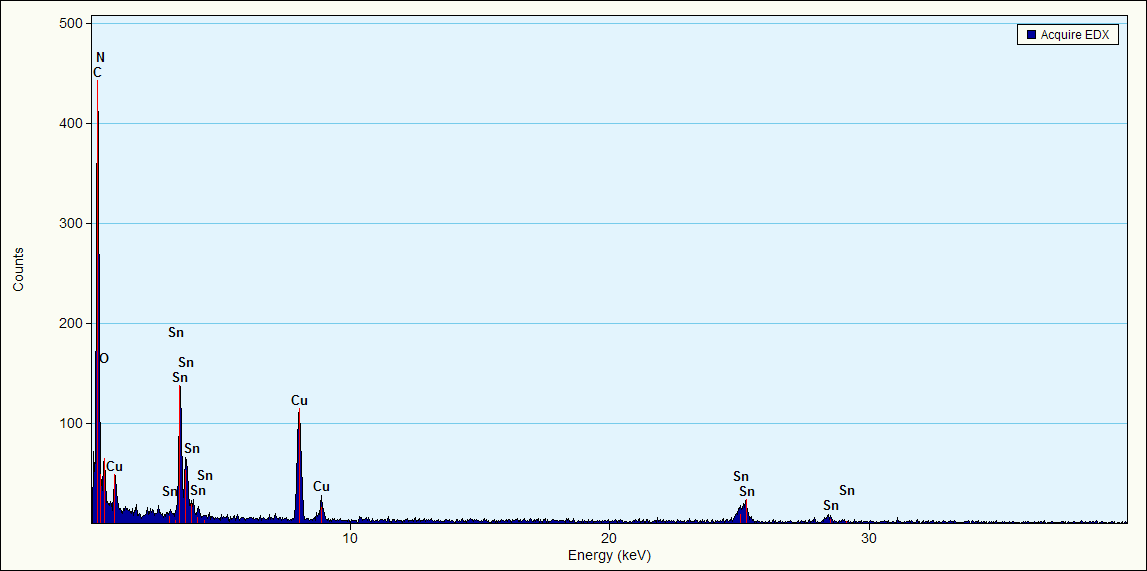


**Figure S11** EDX pattern of Sn@BIBCs.

**
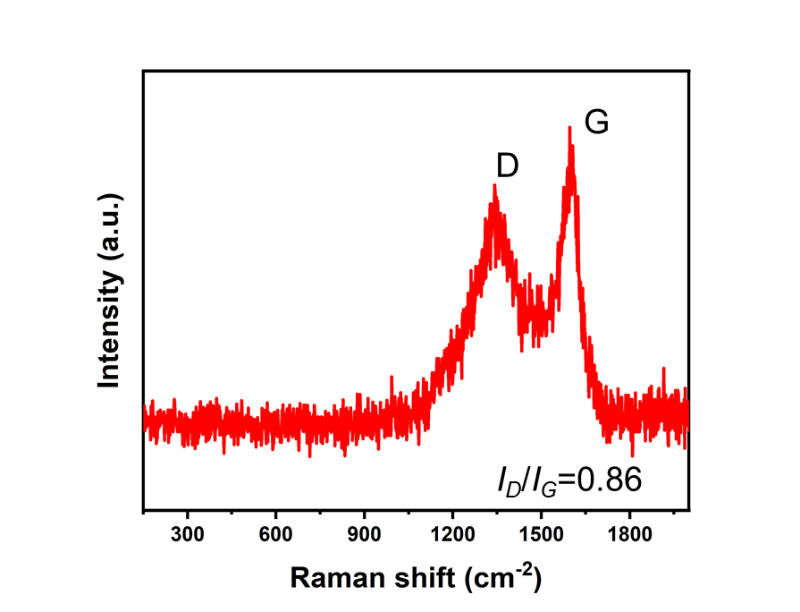
**

**Figure S12** Raman spectrum of Sn@BIBCs

**Note**: The Raman spectra of the Sn@BIBCs composite as illustrated in **Figure S12**, two distinct peaks emerge at 1346 cm^-1^ and 1594 cm^-1^, which are characteristic bands of amorphous carbon.

**
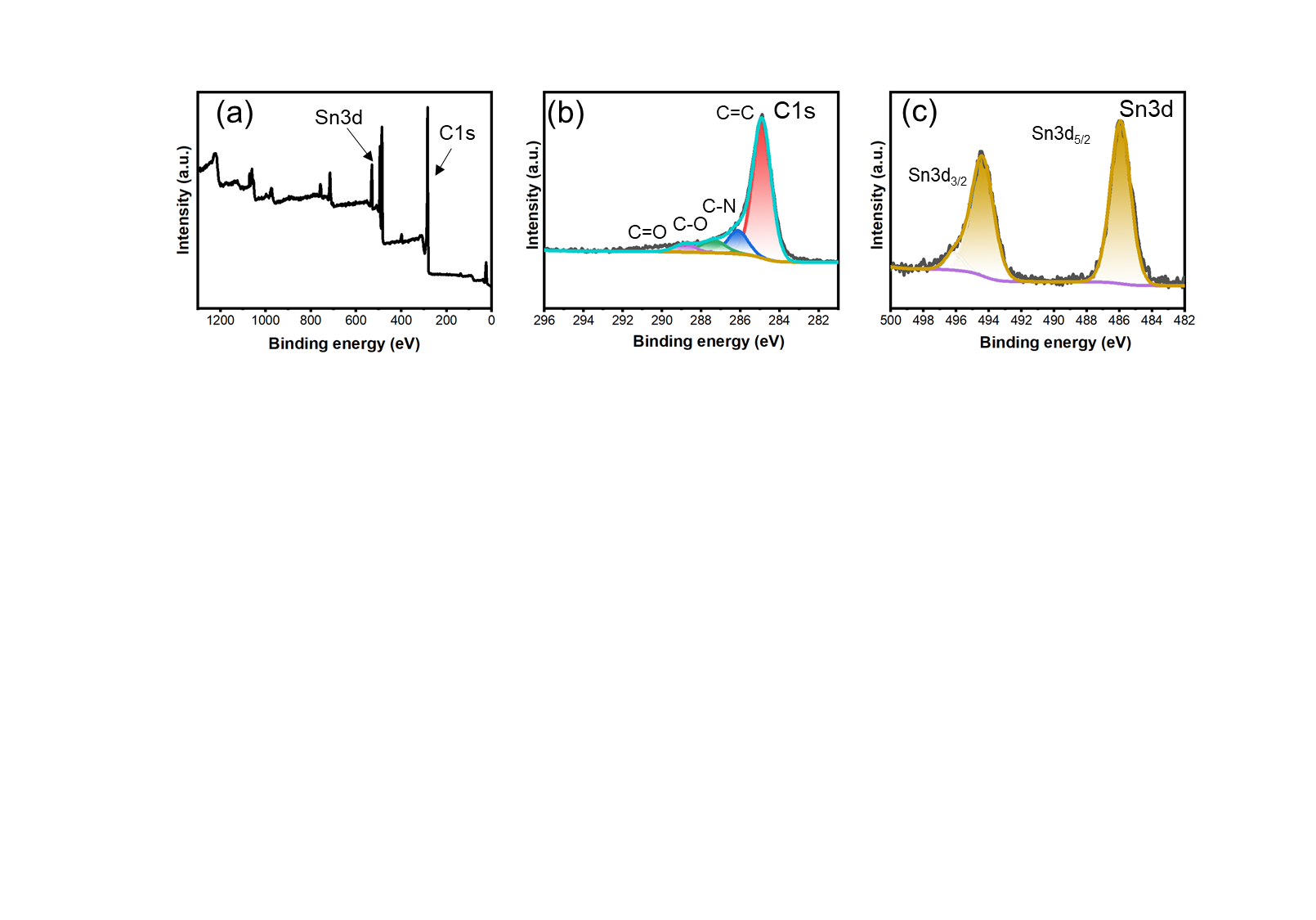
**

**Figure S****13** XPS spectra of Sn@BIBCs: (a) survey (b) C 1s (c) Sn 3d.

**Note**: Surface electronic states within the material were verified using XPS, with the results presented in **Figure S13**. **Figure S13a** shows the survey spectrum of Sn@BIBCs, which reveals the existence of C and Sn. The C1s peaks at 284.3, 285.7, 287.1 and 288.8 eV are assigned to C=C, C–O, C–N and C=O, respectively (**Figure S13b**). In the high-resolution XPS spectrum of Sn 3d (**Figure S13c**), the peaks of 485.8 and 494.3 eV are ascribed to Sn 3d_5/2_ and 3d_3/2_, respectively, confirming the existence of metal Sn.


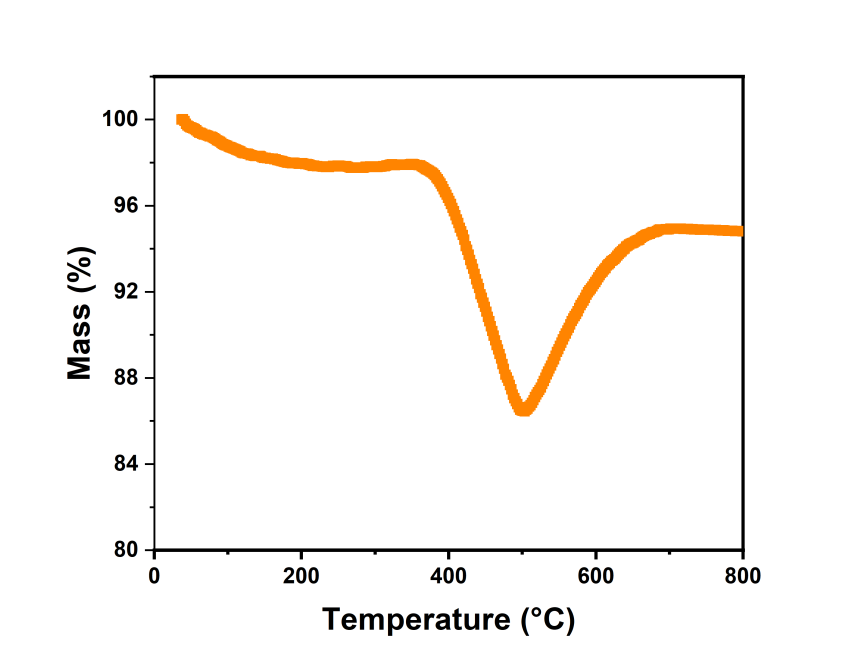


**Figure S14** TGA curve of the Sn@BIBCs.

**Note** :The weight percentage of Sn in the Sn@C composites is calculated according to the following Equation:

*Wt* %=$\frac{\text{ }\text{final}\text{ weight of }\text{S}\text{n}\text{O}\text{2}}{\text{molecular weight of }\text{S}\text{n}\text{O}\text{2}}$ ×$\frac{\text{molecular}\text{ }\text{weight}\text{ }\text{of}\text{ S}\text{n}}{\text{initial}\text{ }\text{weight}\text{ }\text{of}\text{ S}\text{n@}\text{BIBC}}$× 100

=$\frac{\text{ }\text{93.8}}{\text{150.7}}$ × $\frac{\text{118.7}}{\text{100}}$ = 73.8 %

**
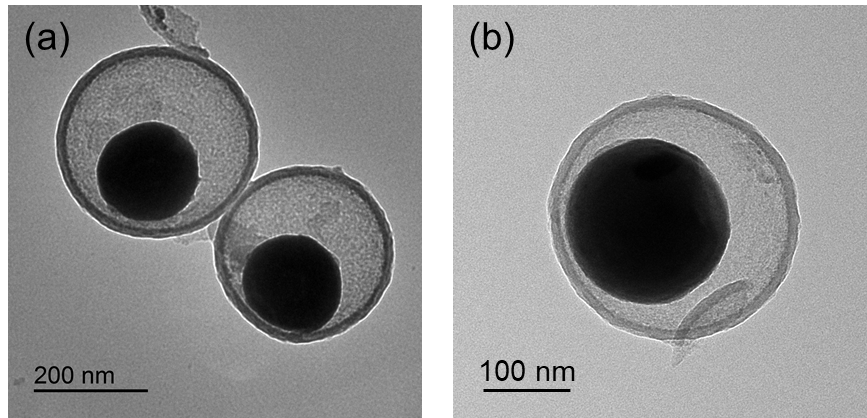
**

**Figure S15** TEM images of Sn@HCSs.

**
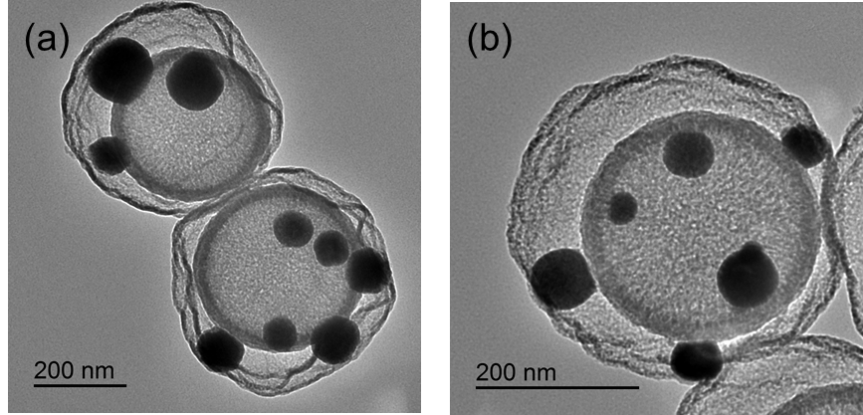
**

**Figure S16** TEM images of Sn@DWHCSs.

**1.4 The characterization of SnSe@BIBCs.**

**
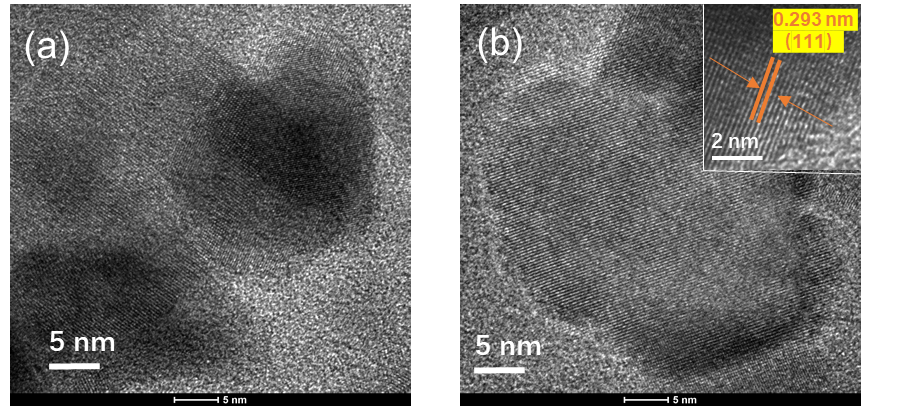
**

**Figure S17** HRTEM images of SnSe@BIBCs.

**
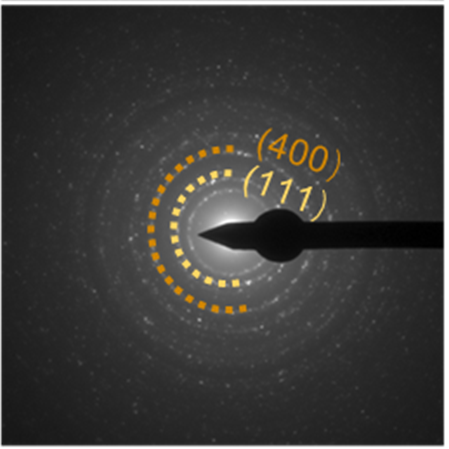
**

**Figure S18** SAED pattern of SnSe@BIBCs.

**
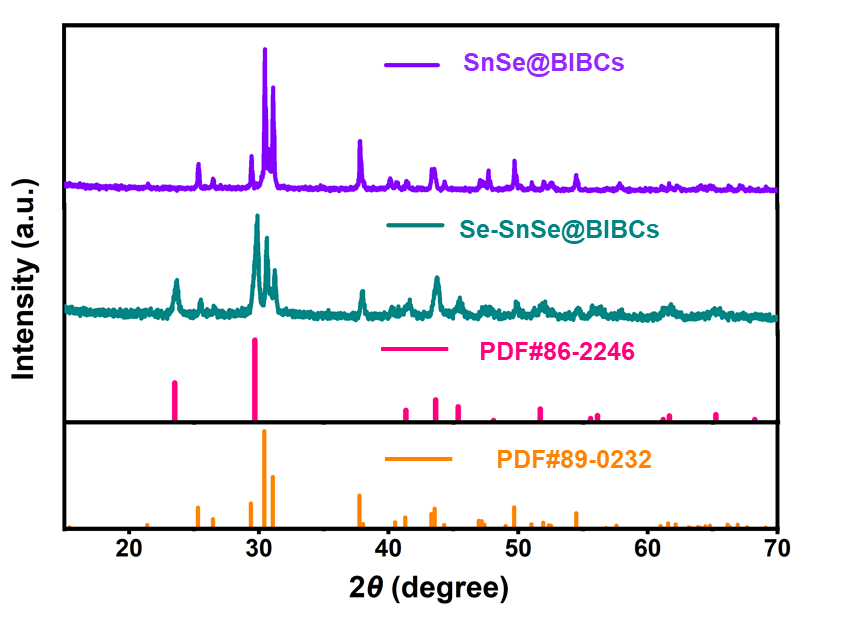
**

**Figure S19** XRD pattern of SnSe@BIBCs and Se-SnSe@BIBCs.

**Note:** HRTEM images display a lamellar structure with sizes roughly tens of nanometers along with crystal faces such as (111), as observed in **Figure S17.** Additionally, the Debye-Scherrer rings of the (111) and (400) planes are attributed to SnSe in the SAED pattern of SnSe@BIBCs (**Figure S18**). The distinguishing peaks of the XRD pattern also match one of the crystal faces stated previously (**Figure S19**).

**
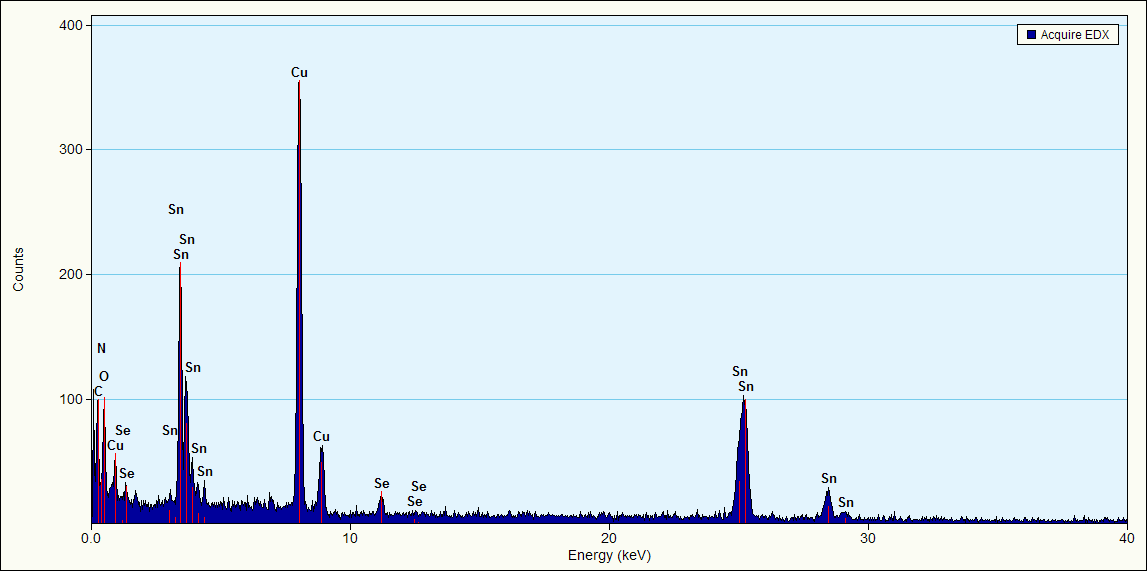
**

**Figure S20** EDX pattern of SnSe@BIBCs.

**
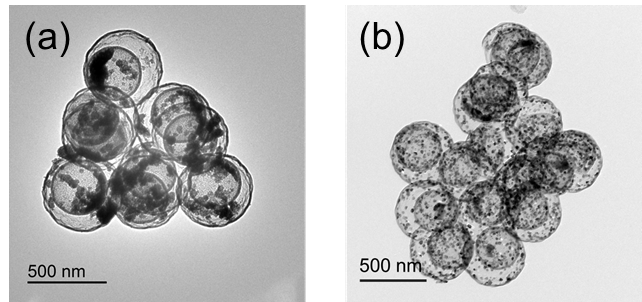
**

**Figure S21** TEM images of (a) SnO_2_@DWHCSs and (b) SnSe@DWHCSs.

**
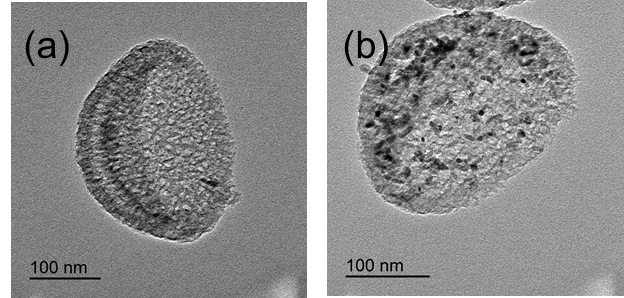
**

**Figure S22** TEM images of (a) Carbon bowls (CBs) (b) SnSe@CBs.

**
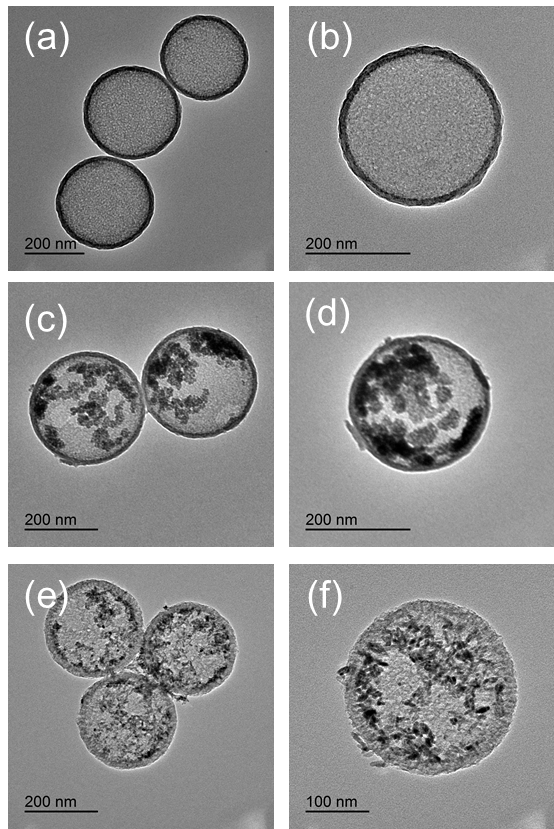
**

**Figure S23** TEM images of (a)(b) hollow carbon spheres (HCSs), (c) (d) SnO_2_@HCSs, (e) (f) SnSe@HCSs.

**1.5 The characterization of Se-SnSe@BIBCs.**


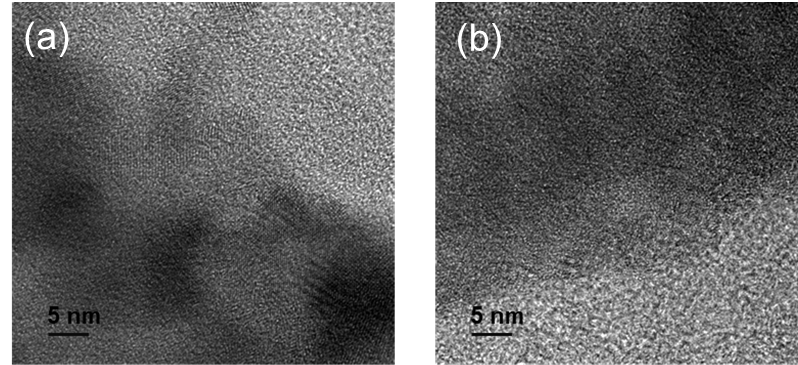


**Figure S24** HRTEM images of Se-SnSe@BIBCs.

**Note**: The Se element observed on the carbon appeared amorphous, providing evidence that selenium was embedded in the mesoporous pores within the carbon wall, in addition to being loaded on the surface of BIBCs.


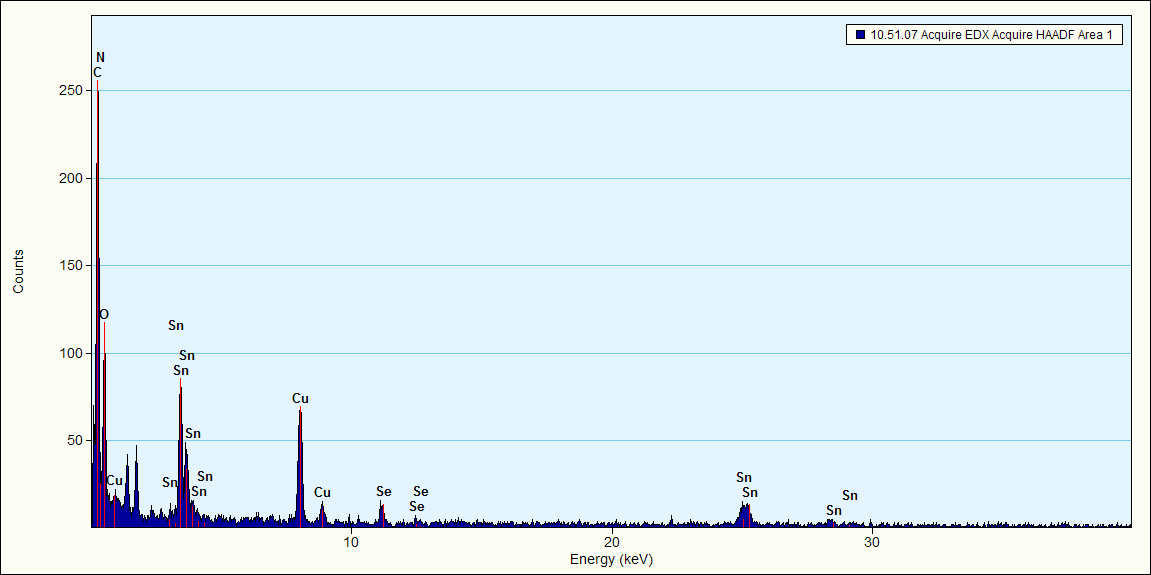


**Figure S25** EDX pattern of Se-SnSe@BIBCs.

**
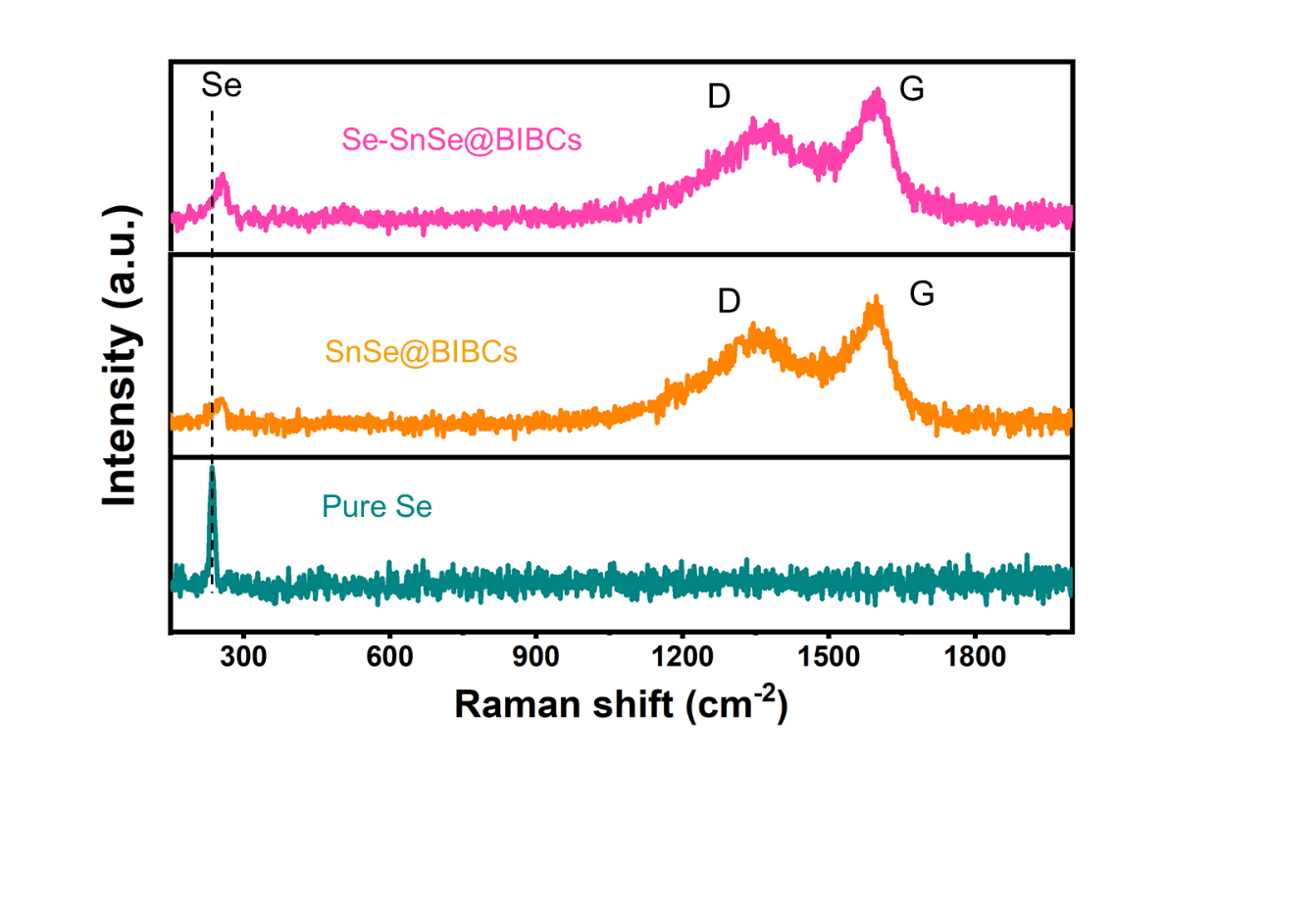
**

**Figure S26** Raman spectra of Se-SnSe@BIBCs, Se@BIBCs and Pure Se.

**Note:** Raman spectroscopy reveals a characteristic 255 cm^−1^ for Se-SnSe@BIBCs compared to commercial Se (237 cm^−1^), indicating confinement of segmental Se in an amorphous state within the pores of the carbon matrix (**Figure S26**).

**
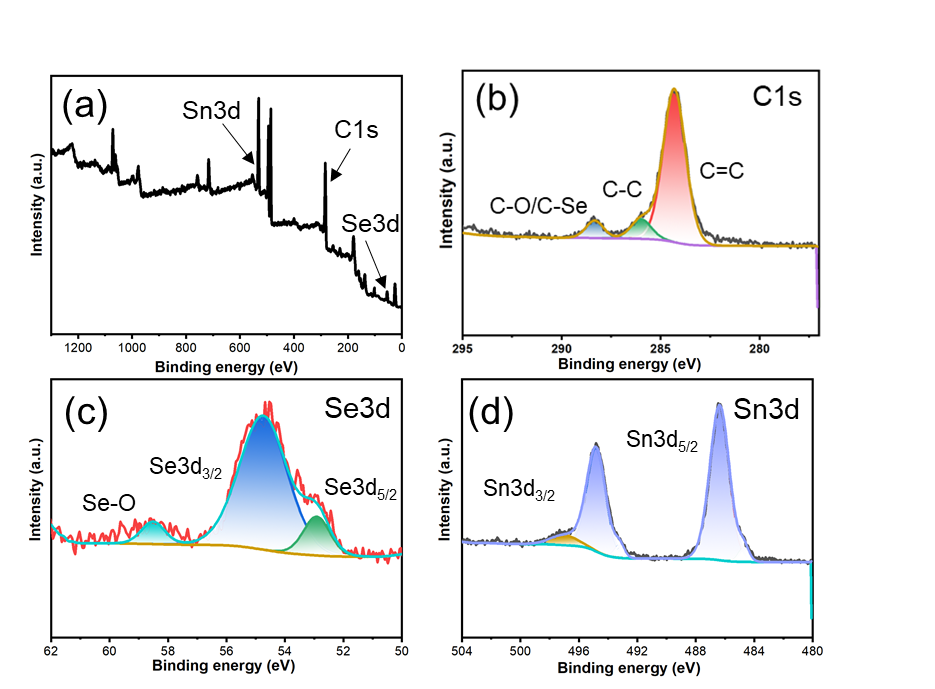
**

**Figure S27** XPS spectra of SnSe@BIBCs: (a) survey, (b) C 1s, (c) Se 3d, and (d) Sn 3d.

**
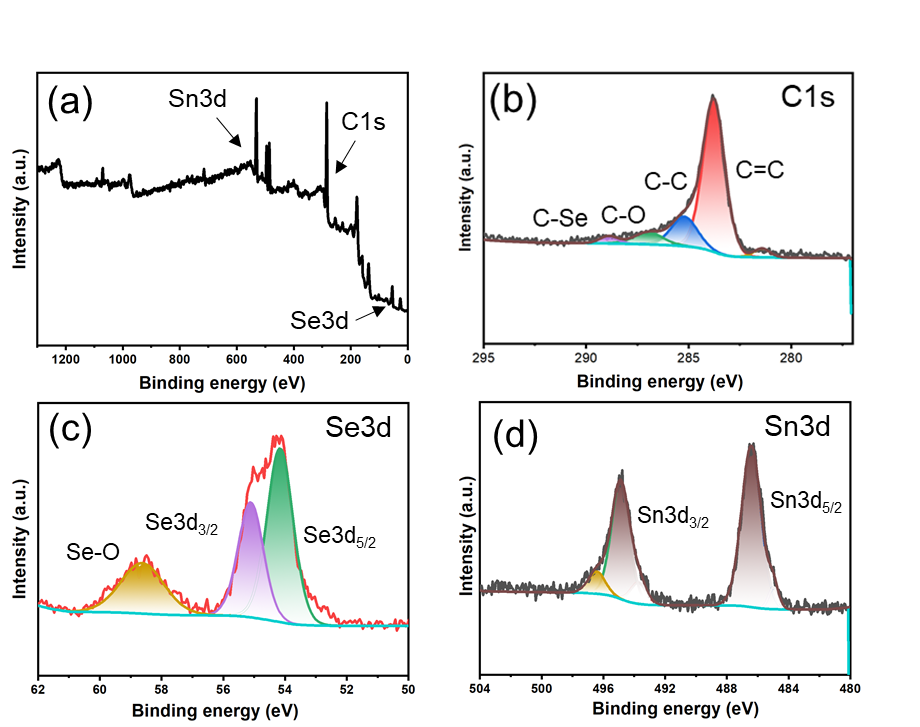
**

**Figure S28** XPS spectra of Se-SnSe@BIBCs: (a) survey, (b) C 1s, (c) Se 3d, and (d) Sn 3d.

**Note：**The XPS spectra before and after loading Se are shown in **Figure S27 and S28.** All samples have similar Sn 3d spectra, with two peaks at about 486.7 and 495.1 eV, which can be attributed to Sn 3d_5/2_ and Sn 3d_3/2_, respectively (Figure S18d and S19d). In SnSe@BIBCs, the C 1s spectrum is deconvoluted into peaks corresponding to C–C (284.8 eV), C–Se/C–O (286.5 eV), and C–O (288.2 eV), respectively. After Se loading, C1s is assigned to peaks corresponding to C-C (284.7eV), C-O (286.8eV), and C-Se (288.3 eV). This illustrates the further loading of Se on carbon and the strong interlayer interaction between C and Se. The spectrum with the biggest change before and after Se loading is undoubtedly Se 3d. Bands at 53.2 eV and 54.7 eV are attributed to the Se3d_5/2_ and Se3d_3/2_ peaks, respectively, shift to 53.9 eV and 55.2 eV. In addition, the ratios of the two peaks are significantly different, which is mainly due to the different proportions of Se and SnSe attributed to Se^0^ and Se^2-^.

**
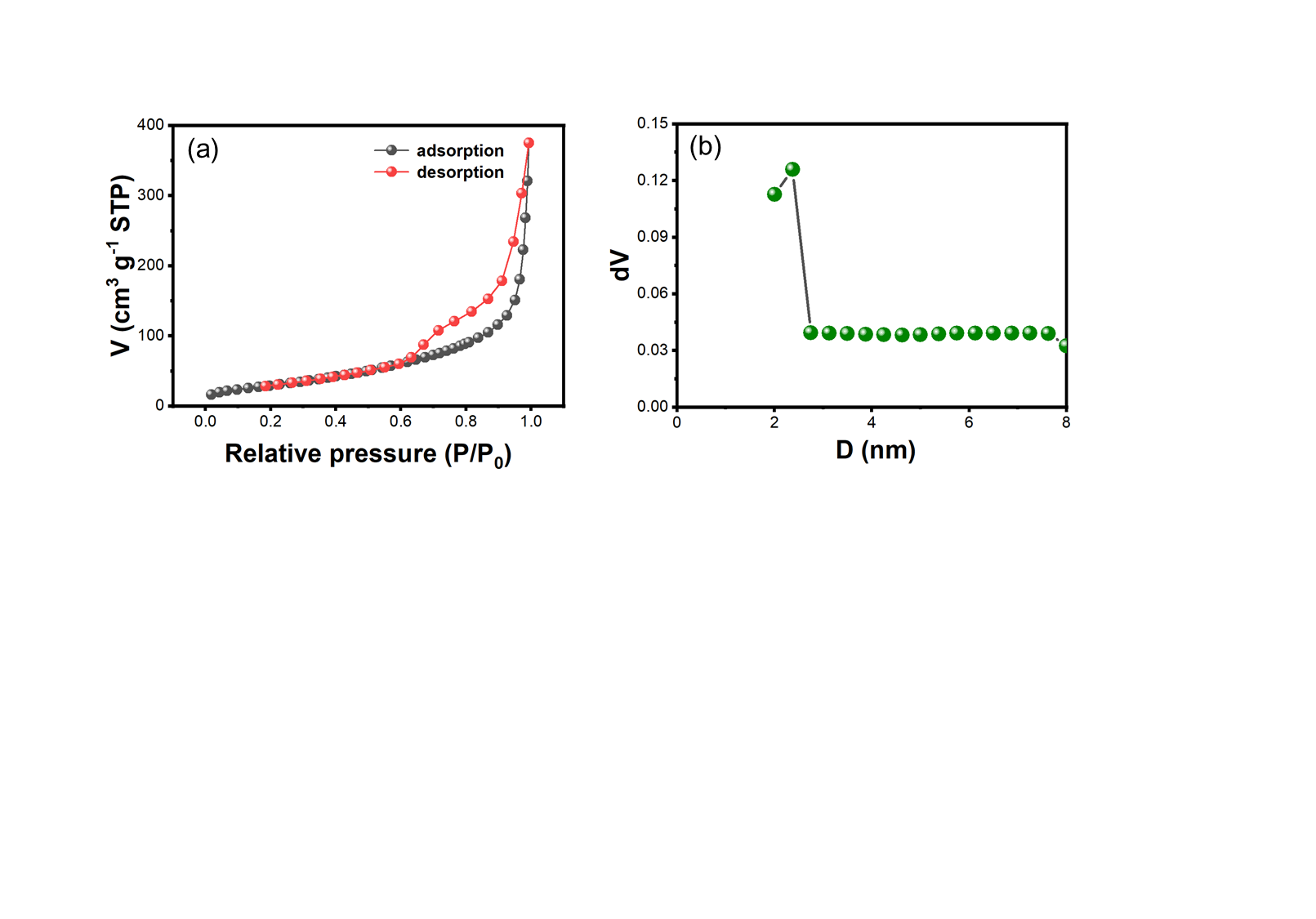
**

**Figure S29** (a) Nitrogen adsorption-desorption isothermal curves and (b) pore size distribution of the Se-SnSe@BIBCs

**Note:** **Figure S29** demonstrates that even after loading selenium and infiltration, the specific surface area of Se-SnSe@BIBCs remains high (344.7 m^2^ g^−1^).

**
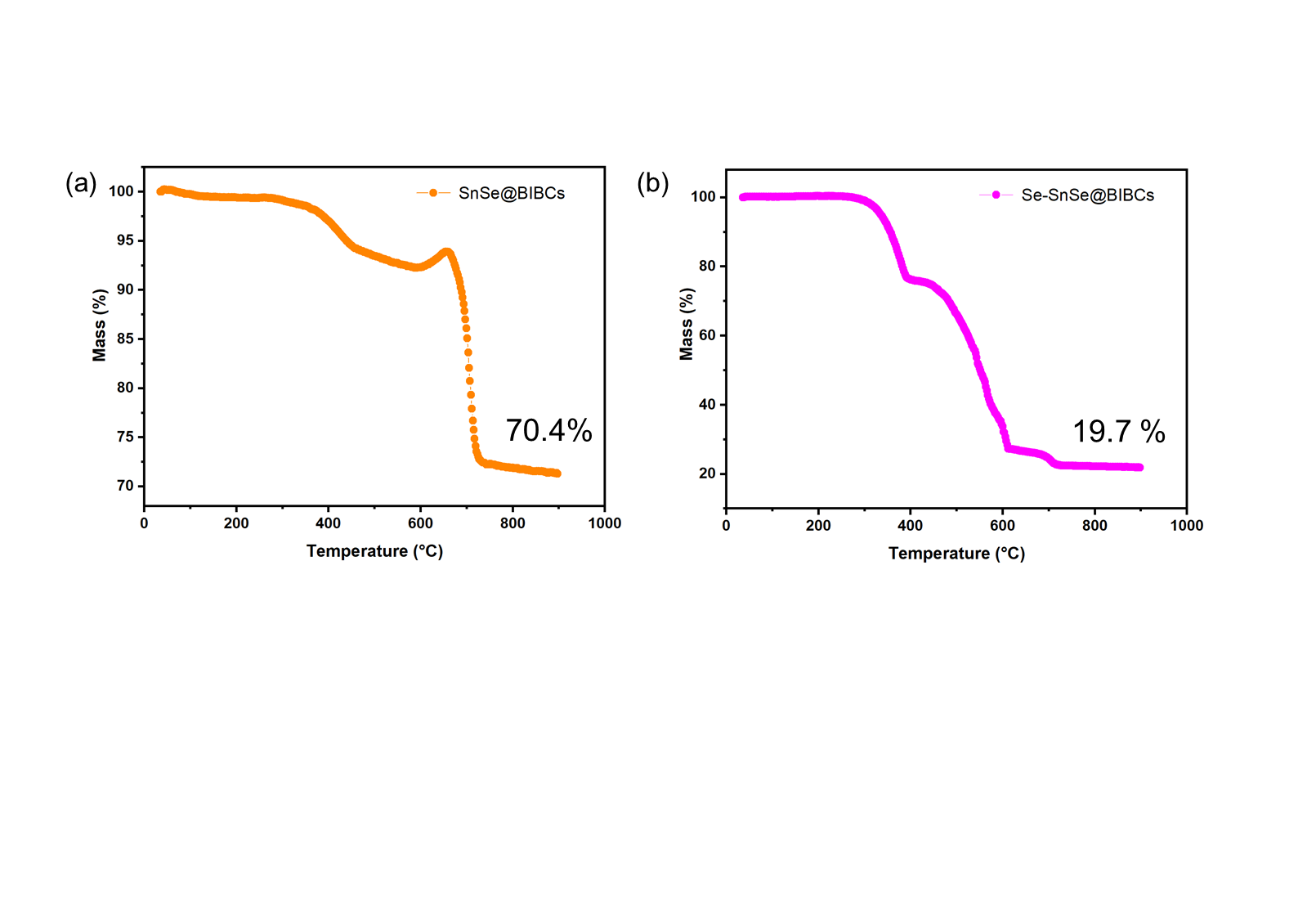
**

**Figure S30** TGA curve of the SnSe@BIBCs.

**
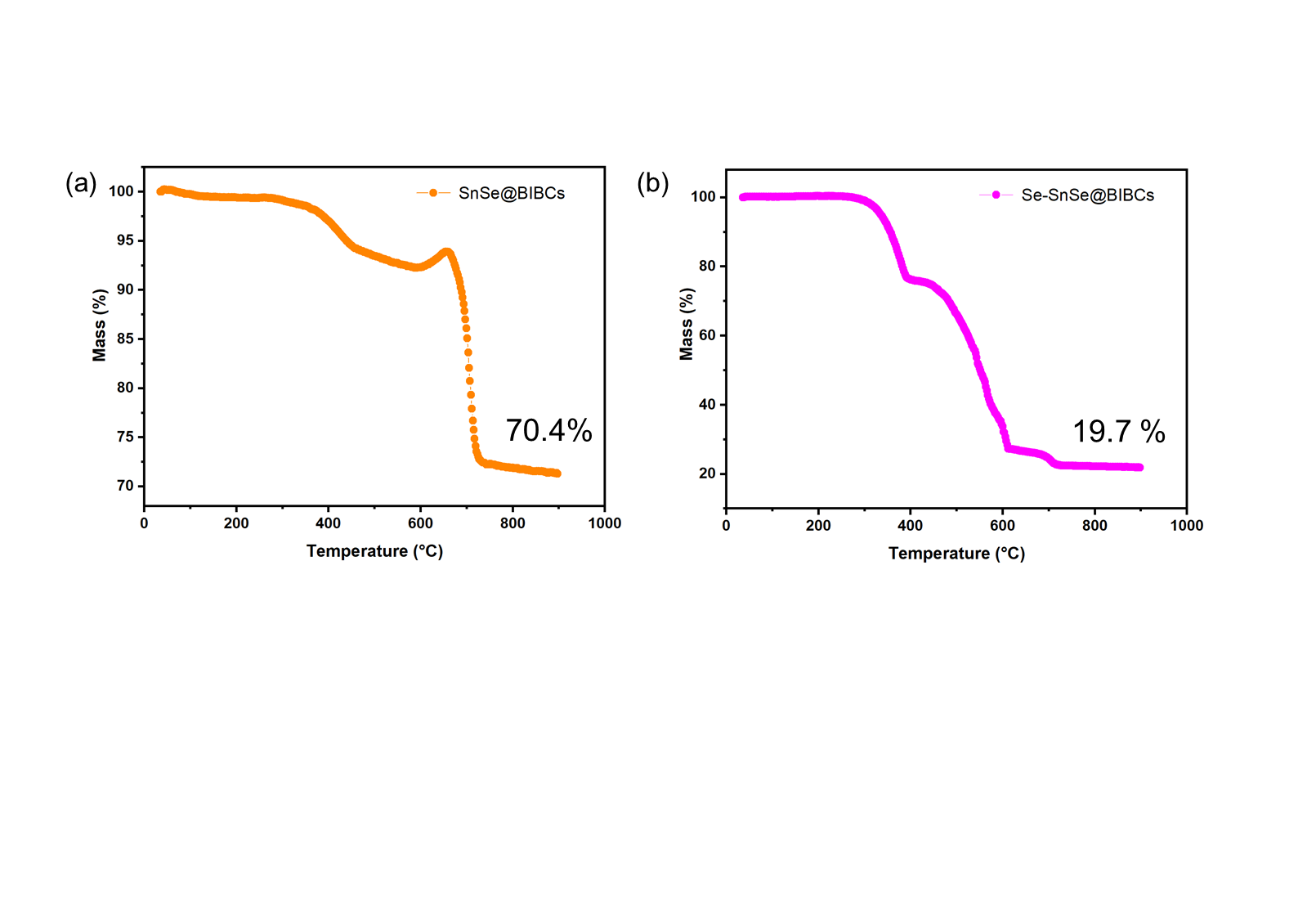
**

**Figure S31** TGA curve of Se-SnSe@BIBCs.

**Note:** $Weight of Sn\mathrm{Se}=weight of \mathrm{Sn}O_{2}\times\frac{Molecular weight of SnSe}{Molecular weight of SnO_{2}}=74.4\%$ (Equation 1).

To determine the weight content of SnSe and carbon in the composite, thermogravimetric analysis (TGA) was performed from room temperature to 900 °C at a heating rate of 5 °C min^−1^ in an oxygen atmosphere. **Figure S30** manifests TGA curves of SnSe@BIBCs. A weight loss occurs between 400 and 600 °C, probably due to oxidation of SnSe and volatilization of SeO_2_: SnSe+O_2_=SnO_2_ +SeO_2_. Then, a second weight drop occurs between 650 and 900 °C, which is related to the combustion of carbon in the composite. Finally, when the temperature reached 900 °C, the tin oxide residue still accounted for 56.7 *wt*% of the total mass. Therefore, the content of SnSe in the composite is 74.4 *wt*%, while the content of carbon is 25.6 *wt*%.

The TGA curve at Se-SnSe@BIBCs also shows the weight loss between 400 and 600 ° C due to volatilization of SeO_2_ :Se+O_2_=SeO_2_, with the remaining 19.7% being the weight ratio of SnO_2_. According to the above algorithm (Equation 1), SnSe is 25.8 *wt* % and C is 8.86 *wt* %, so the weight ratio of Se is 65.4% (**Figure S31**).

**
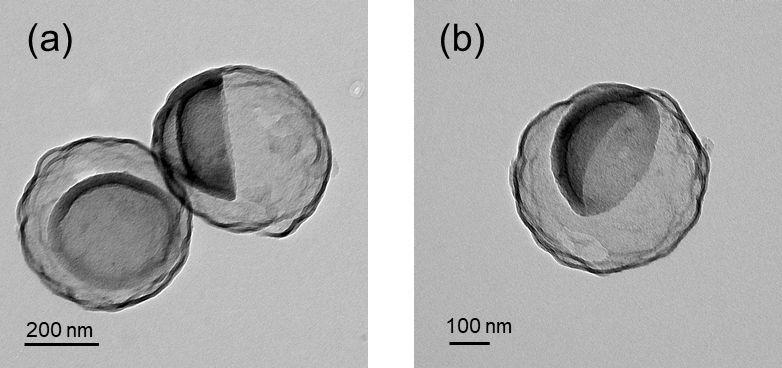
**

**Figure S32** TEM images of Se-BIBCs.

**
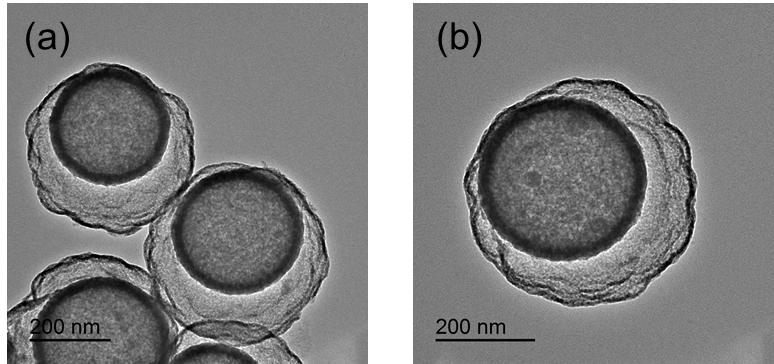
**

**Figure S33** TEM images of Se-DWHCSs.

**
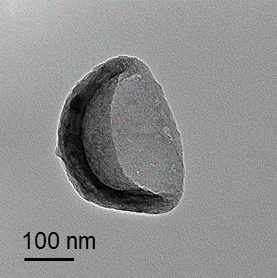
**

**Figure S34** TEM image of Se-carbon bowl.

**1.6 Supplementary data on the performance of Se-SnSe@BIBCs as cathode materials.**

**
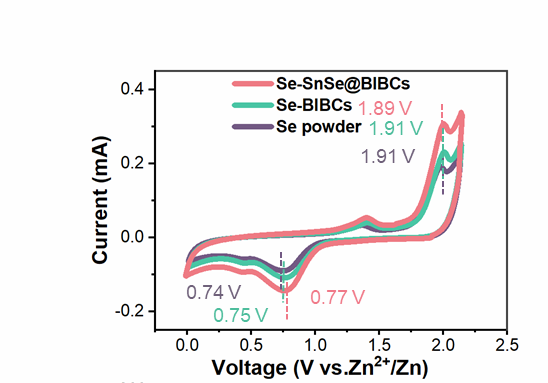
**

**Figure S35** Comparison of CV curves of Se-SnSe@BIBCs, Se-BIBCs and Se powder electrodes.


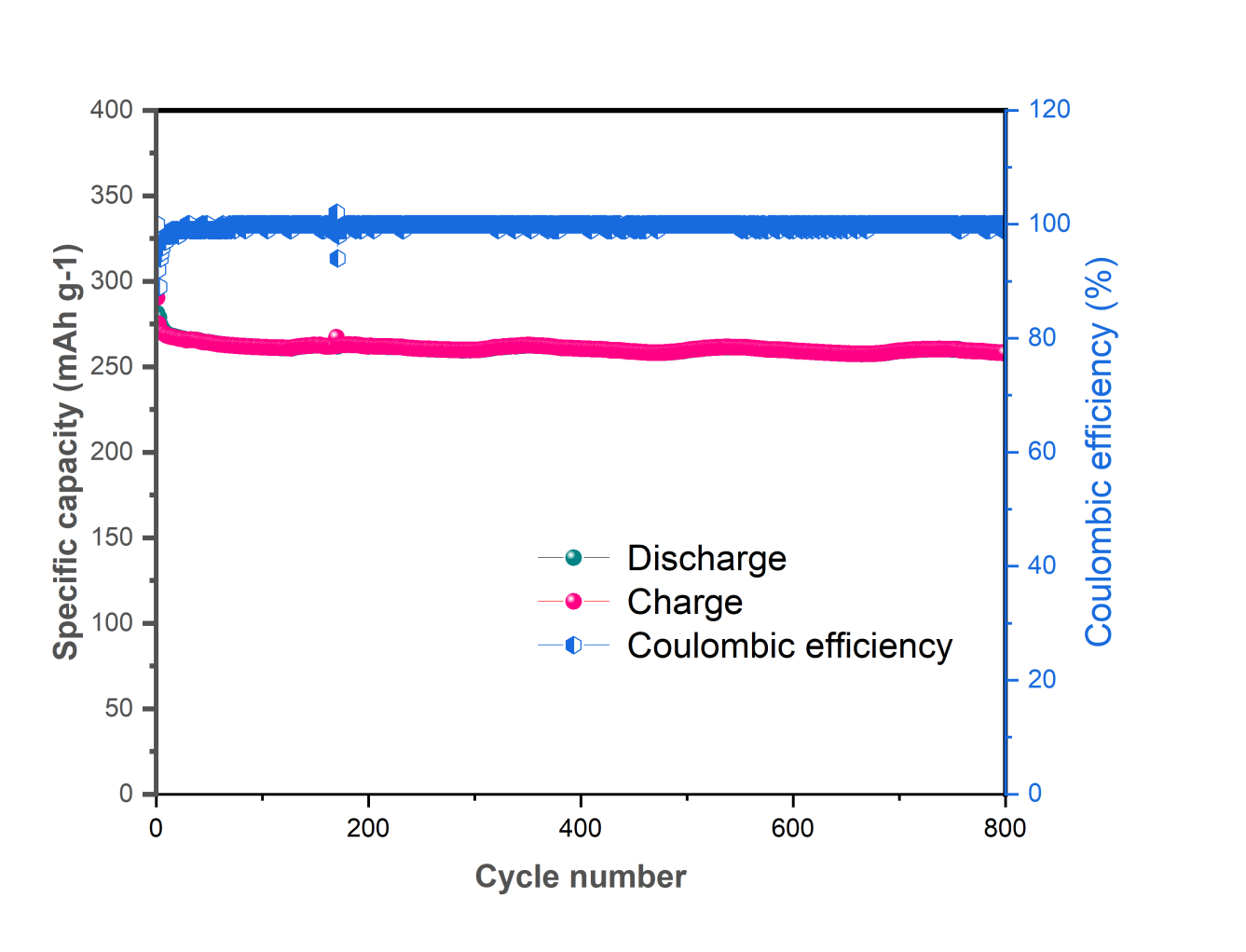


**Figure S36** Cycling performances of Se-SnSe@BIBCs at 5 A g^-1^.

**1.7 DFT theoretical calculations and *in situ* characterization of Se-SnSe@BIBCs when used as cathode materials.**

**
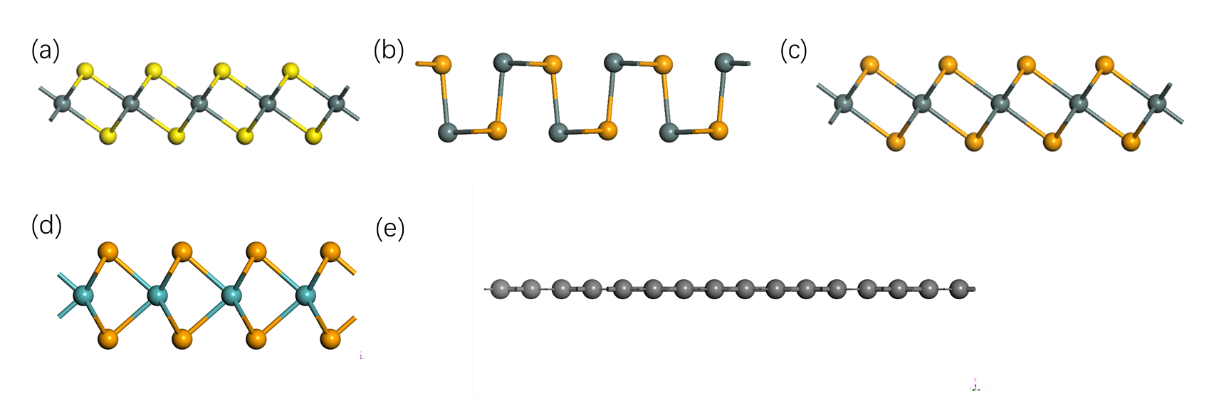
**

**Figure S37** Side view of (a) SnS_2_, (b) SnSe, (c) SnSe_2_, (d) MoSe_2_ and (e) graphene

**
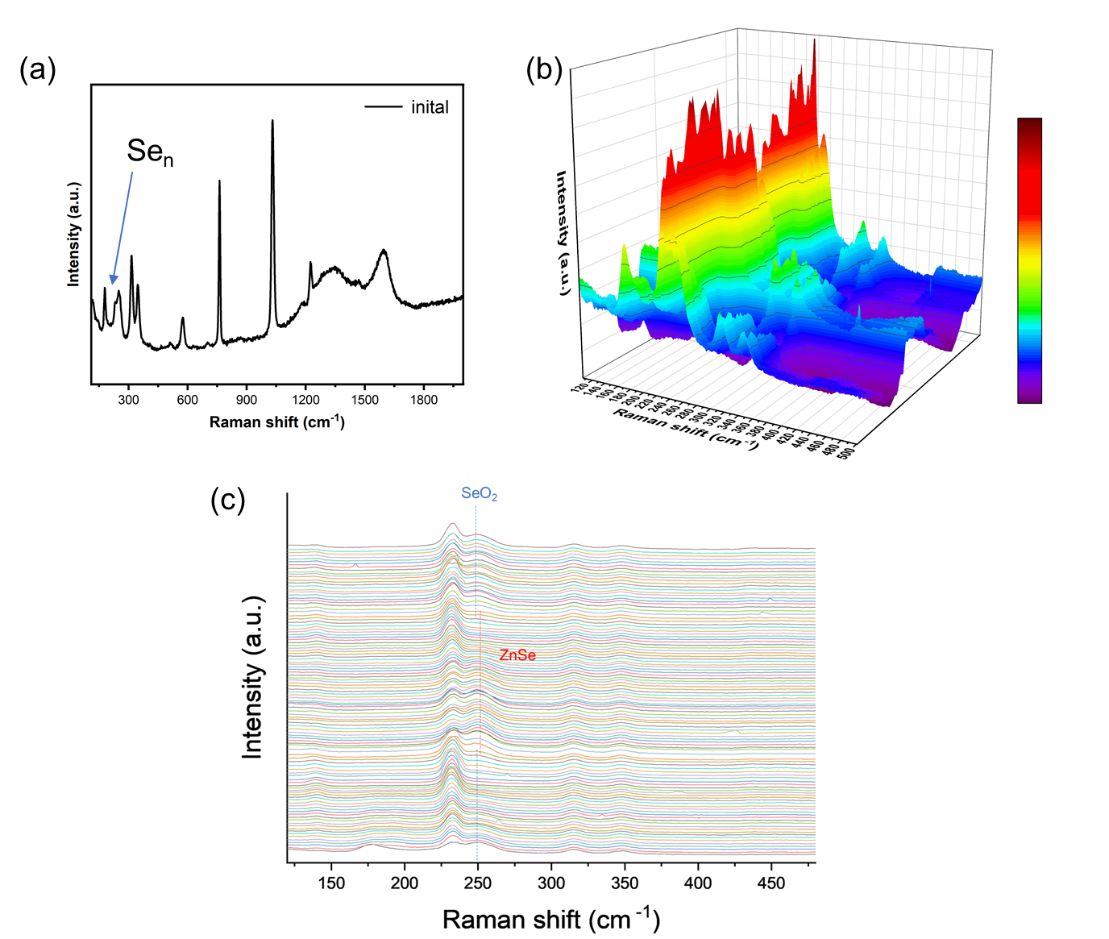
**

**Figure S38** (a) Raman spectrum of initial electrolyte, (b-c) *in situ* Raman spectra at the first cycle.


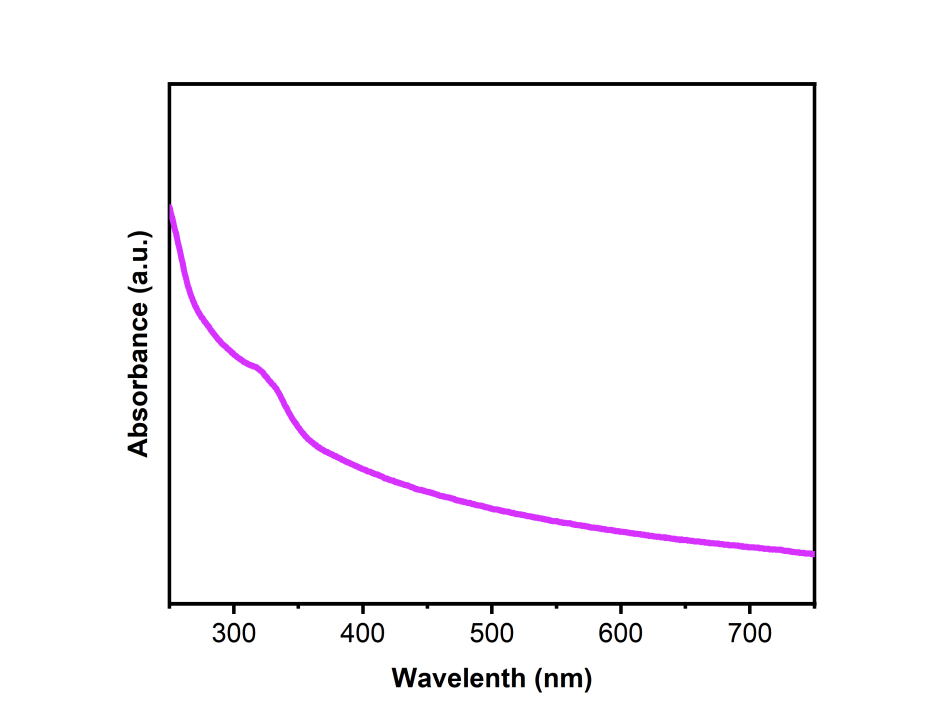


**Figure S39** UV−vis absorption spectrum of initial electrolyte.

**
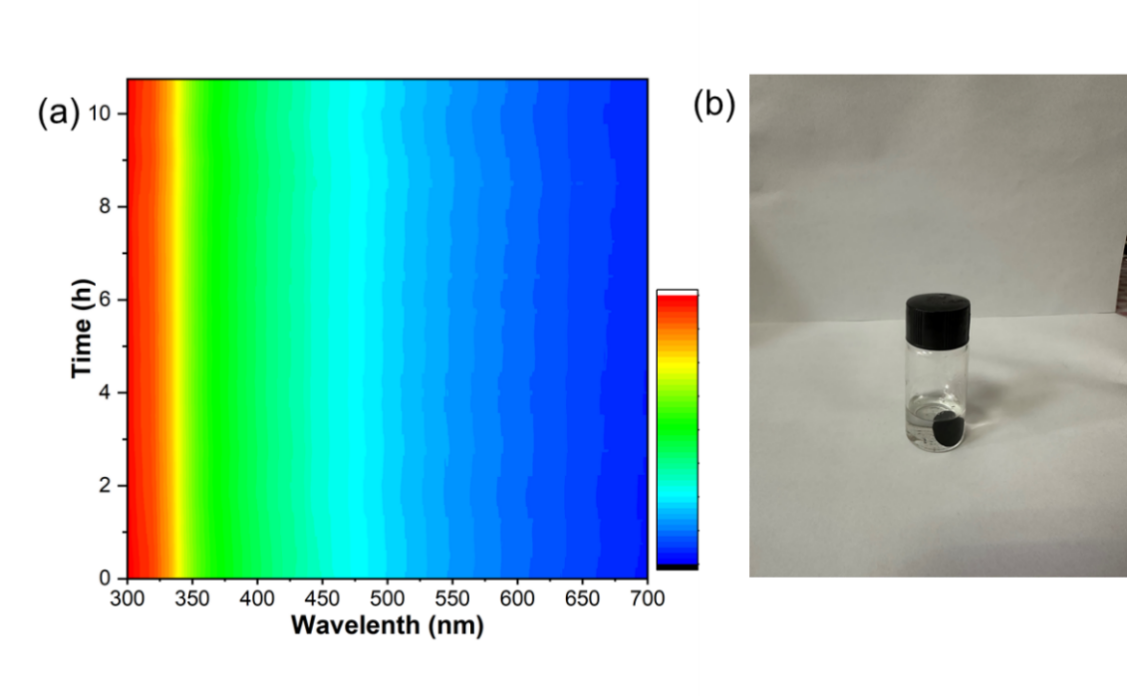
**

**Figure S40** *In situ* UV spectra of electrolyte in static state.

**1.8 Supplementary electrochemical properties of Sn@BIBCs-Zn metal anodes.**

**
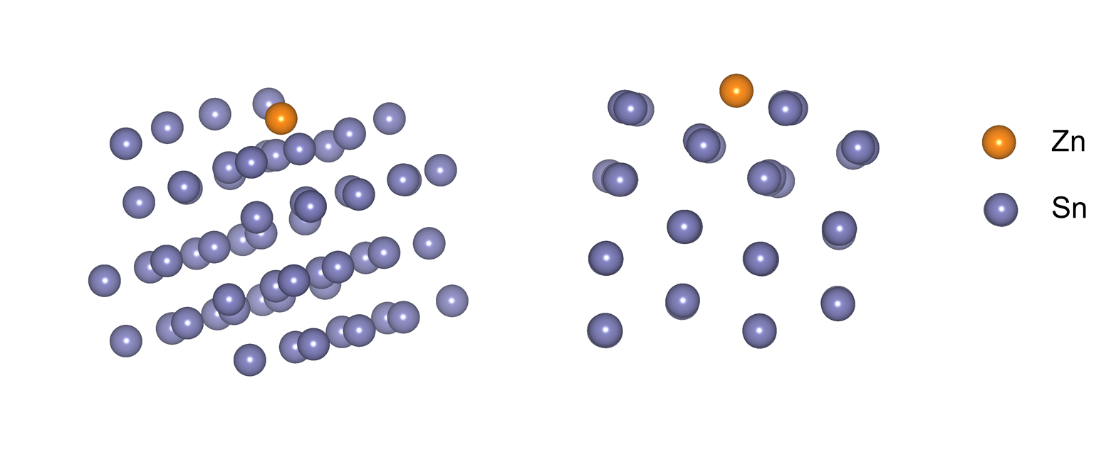
**

**Figure S41** Side view of Sn and Zn models in DFT calculation.

**
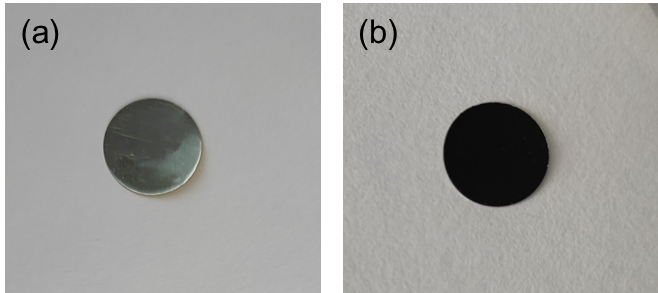
**

**Figure S42** Optical photo images of (a) zinc foil and (b) Sn@BIBCs-Zn.

**
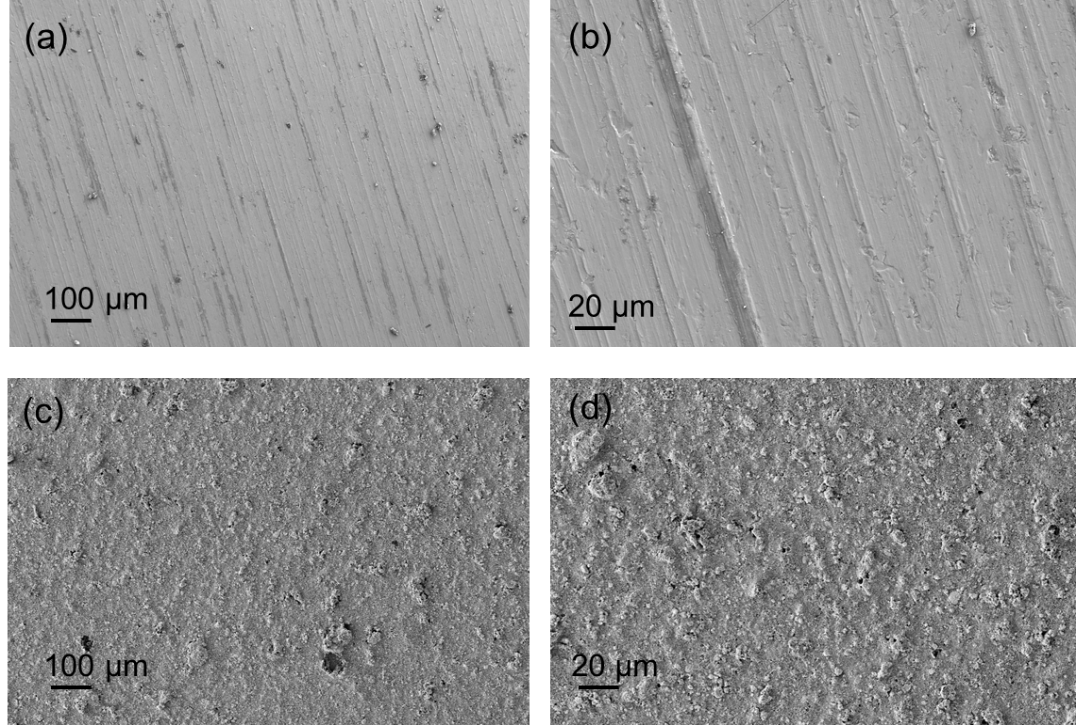
**

**Figure S43** SEM images of (a-b) zinc foil and (c-d) Sn@BIBCs-Zn.


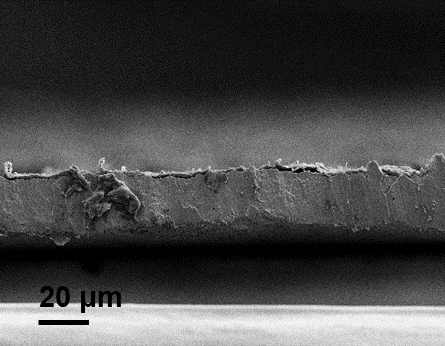


**Figure S44** Cross-sectional images of Sn@BIBCs-Zn


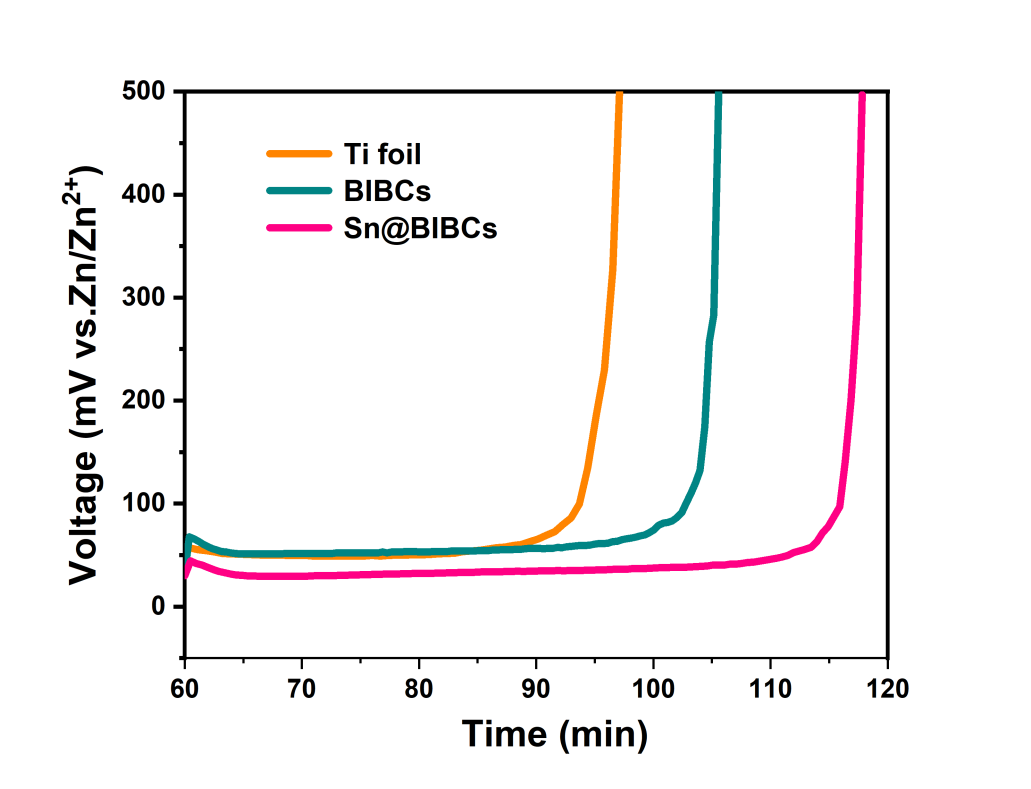


**Figure S45** Voltage profiles of Zn stripping from Ti foil, BIBCs and Sn@BIBCs.

**
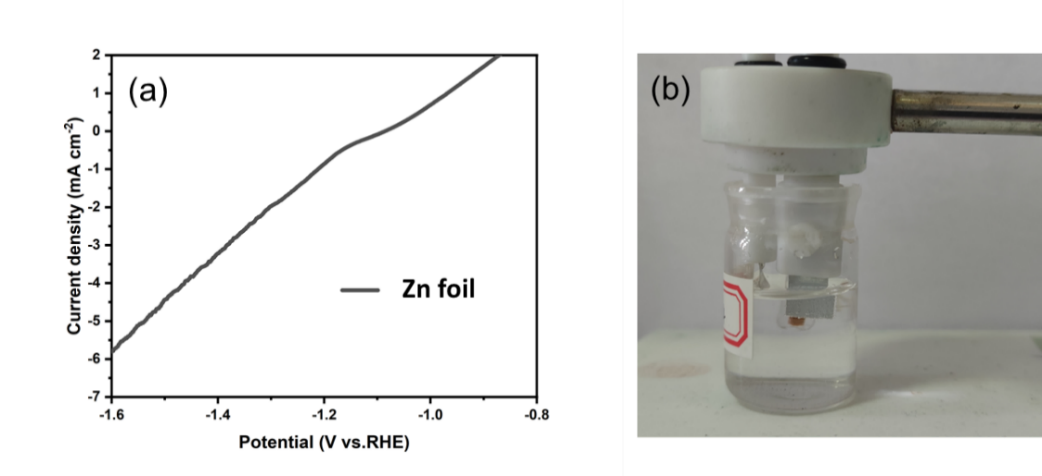
**

**Figure S46** (a) LSV curve for HER and (b) photograph of Zn foil in ZnSO_4_.


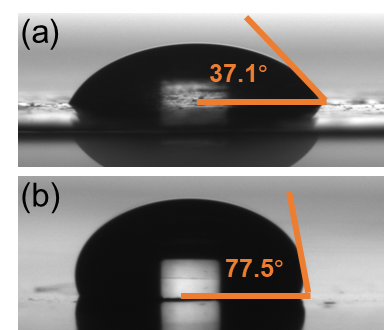


**Figure S47** Wettability tests using ZnSO_4_ electrolyte on (a) Sn@BIBCs-Zn and (b) Zn foil.

**
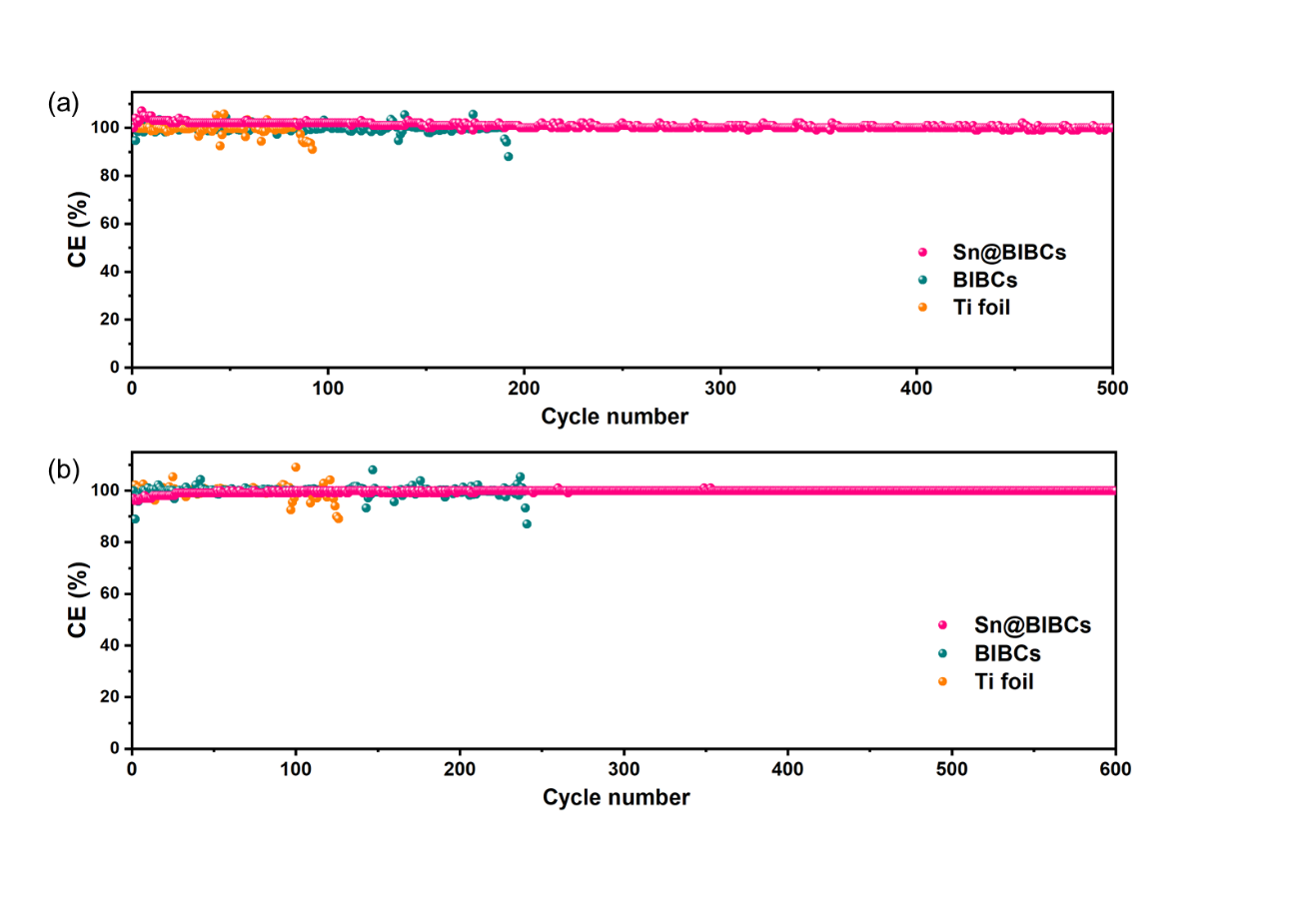
**

**Figure S48** CE plots of different electrodes at 5 mA cm^-2^ at a capacity of (a) 1 mAh cm^-2^ and (b) 2 mAh cm^-2^.

**
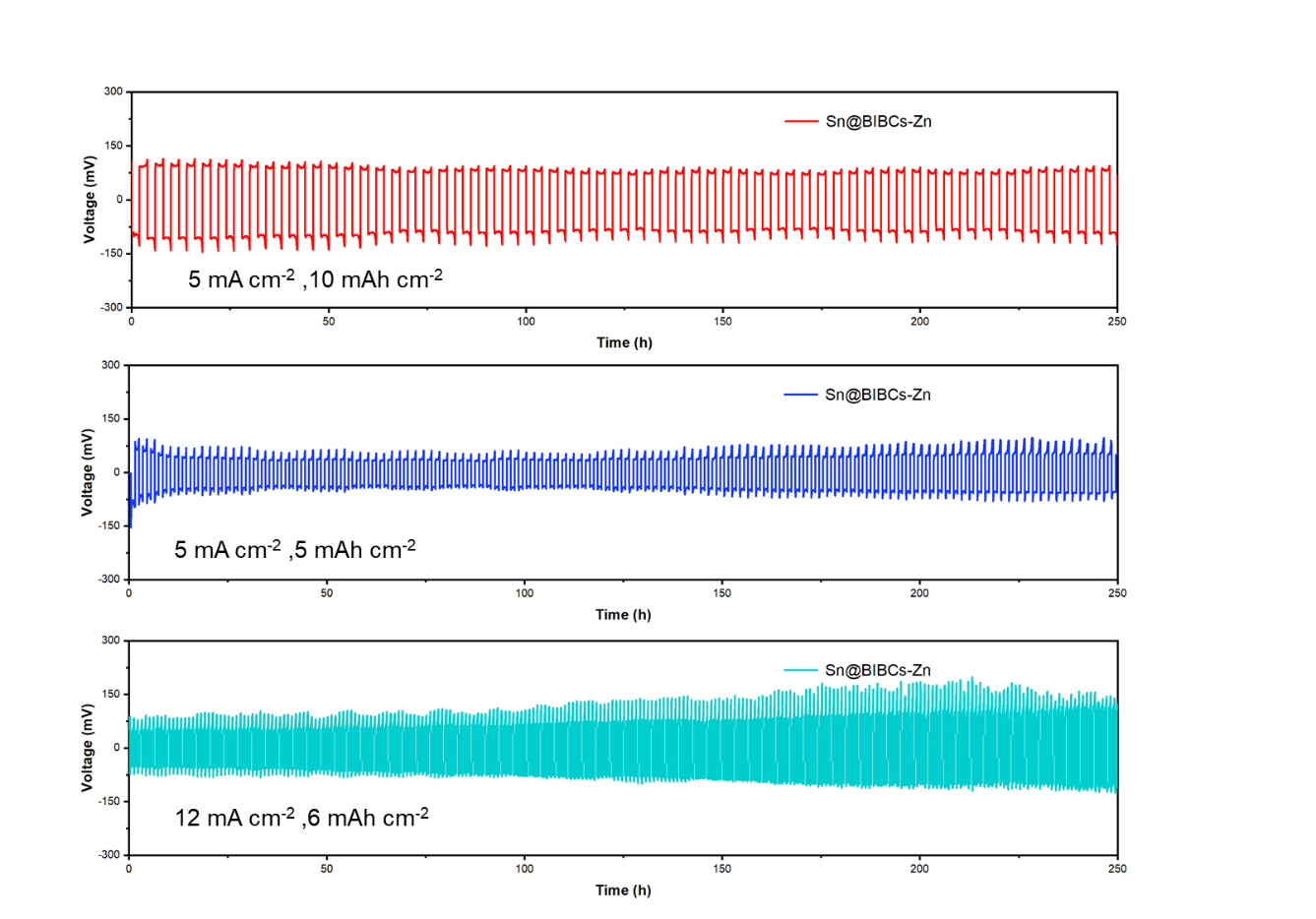
**

**Figure S49** Cycling performances of symmetric cells (a) at 5 mA cm^-2^ for 10 mAh cm^-2^, (b) at 5 mA cm^-2^ for 5 mAh cm^-2^, and (c) 12 mA cm^-2^ for 6 mAh cm^-2^ using Sn@BIBCs-Zn electrode with a Zn plating capacity.

**1.9 Conformational changes of Sn@BIBCs-Zn metal anodes after electrochemical reaction.**

**
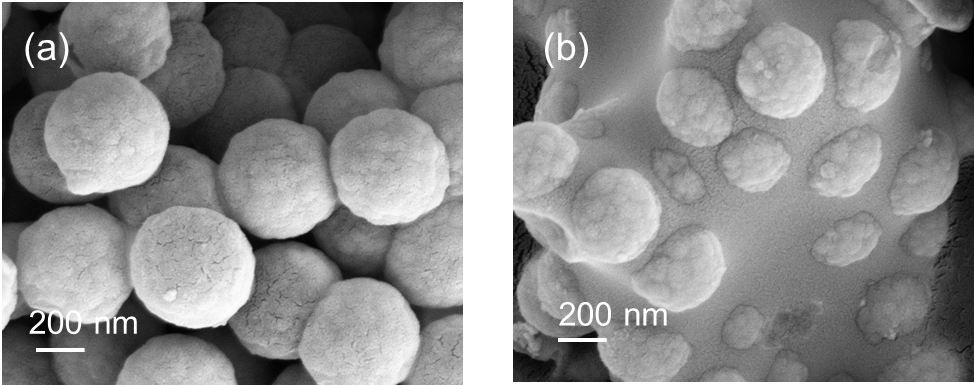
**

**Figure S50** Zn deposition behaviors on the Sn@BIBCs (a) before and (b) after Zn plating for 2 mAh cm^-2^.

**
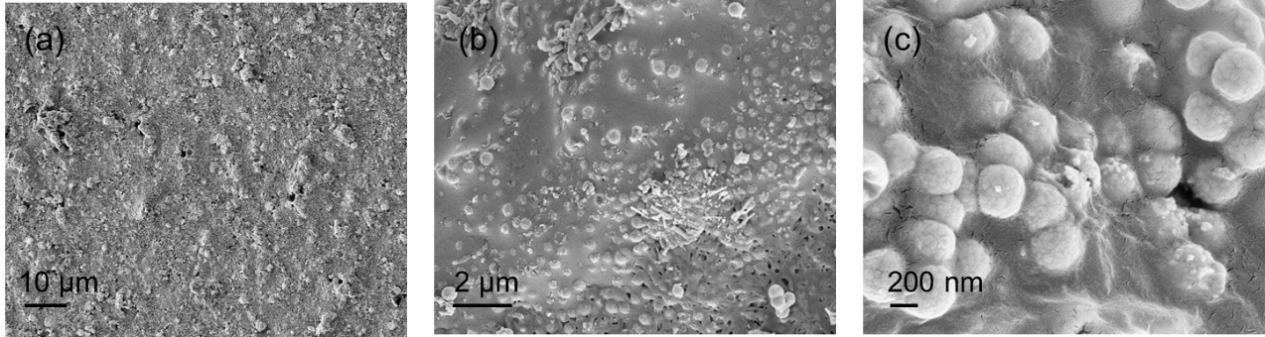
**

**Figure S51** SEM images with different magnification of Zn deposition behaviors on the Sn@BIBCs for 5 mAh cm^-2^.

**
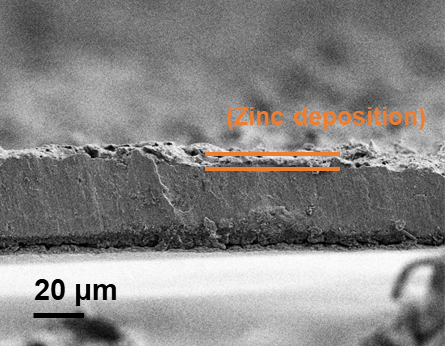
**

**Figure S52** Cross-sectional images of Zn deposition behaviors on the Sn@BIBCs for 5 mAh cm^-2^.


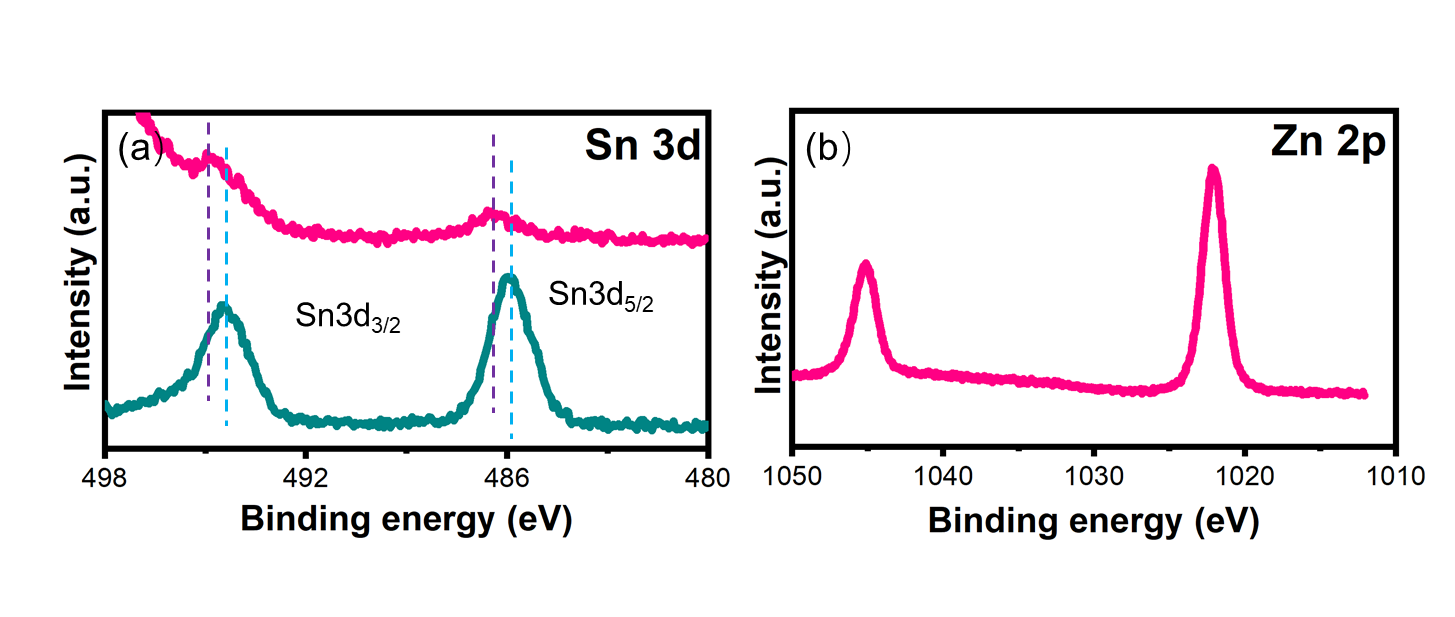


**Figure S53** XPS spectra of Sn@ BIBCs during Zn plating. XPS spectra of (a) Sn 3d before and after Zn plating and (b) Zn 2p after Zn deposition.

**Note：**The XPS spectra before and after galvanization showed that the Sn3d 5/2 and Sn 3/2 spectra moved from 485.8 eV and 494.5 eV to 486.4 eV and 495.1 eV, respectively**.**

**
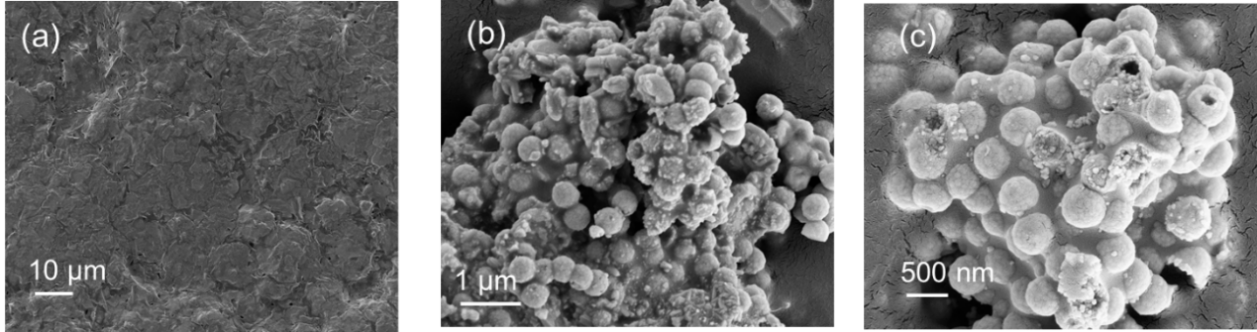
**

**Figure S54** SEM image with different magnification of the Sn@BIBCs-Zn after stripping with a capacity of 5 mAh cm^-2^.

**
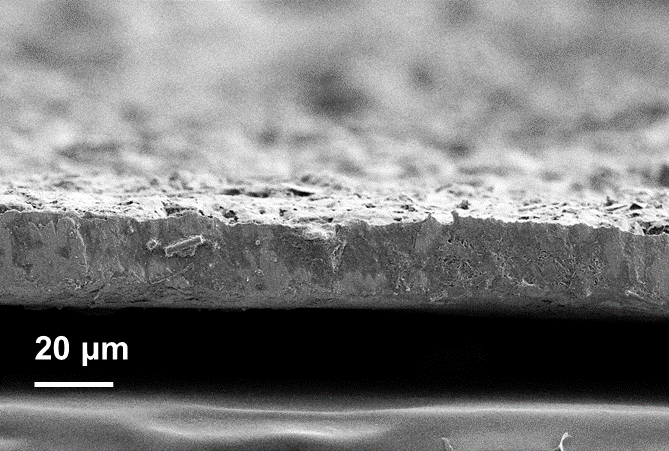
**

**Figure S55** Cross-sectional images of the Sn@BIBCs-Zn after stripping with a capacity of 5 mAh cm^-2^.

**
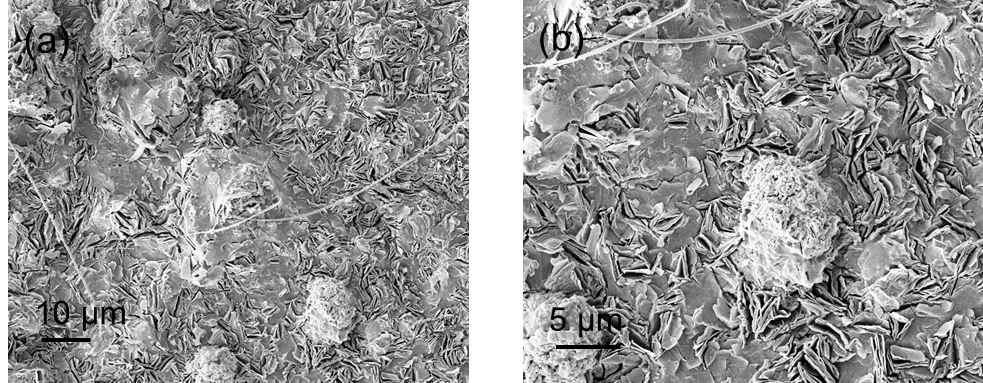
**

**Figure S5****6** Zn deposition behavior on Zn foil. SEM images with different magnification of the surfaces of Zn foil for 5 mAh cm^-2^.

**
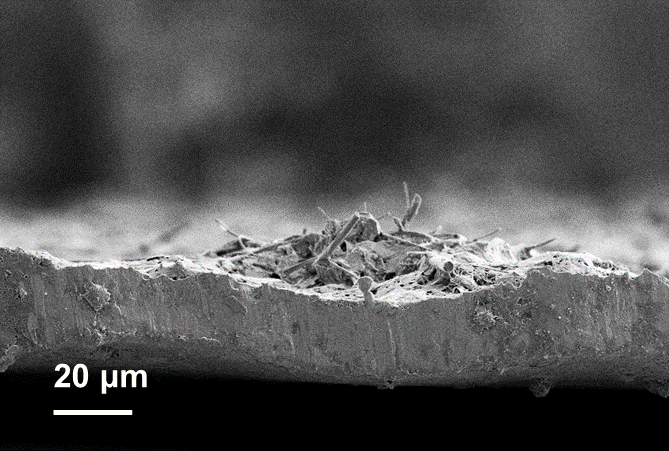
**

**Figure S57** Cross-sectional images of Zn deposition behaviors on Zn foil for 5 mAh cm^-2^.

**
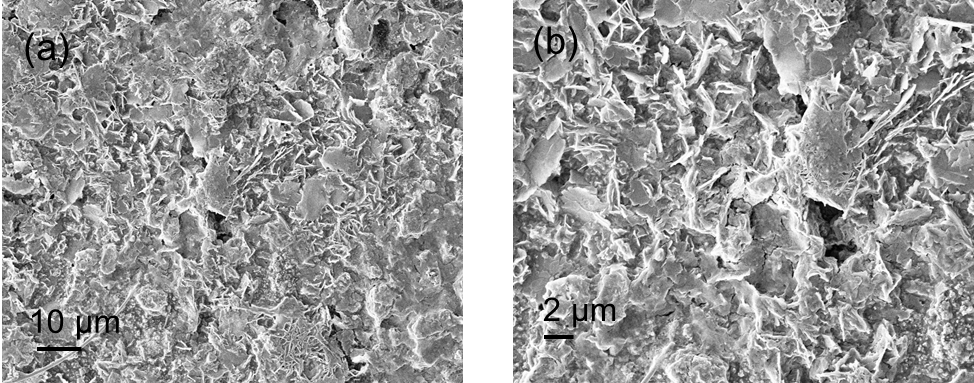
**

**Figure S58** Zn stripping behavior on Zn foil. SEM images with different magnification of the surface of Zn foil for 5 mAh cm^-2^.


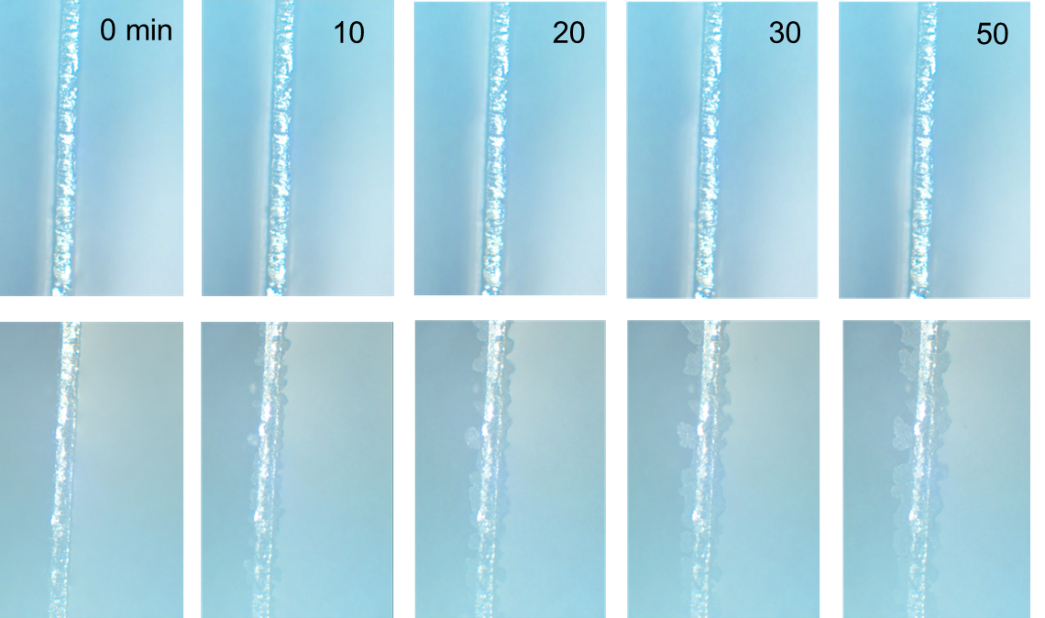


**Figure S59** In situ optical observations of the Zn plating process on (a) Sn@BIBCs-Zn and (b) Zn foil as a function of time at a current density of 10 mA cm^−2^.

**
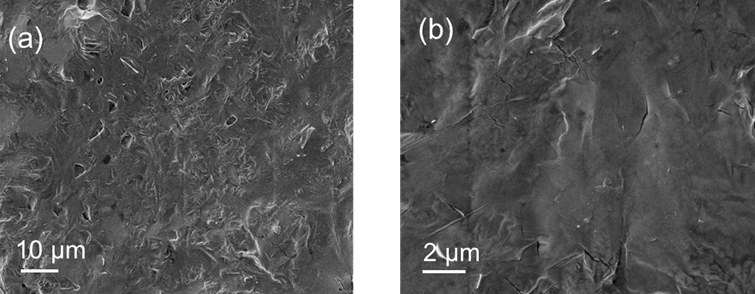
**

**Figure S60** SEM images with different magnification of Sn@BIBCs-Zn plating/stripping at a current density of 1 mA cm^−2^ on 20 h.

**
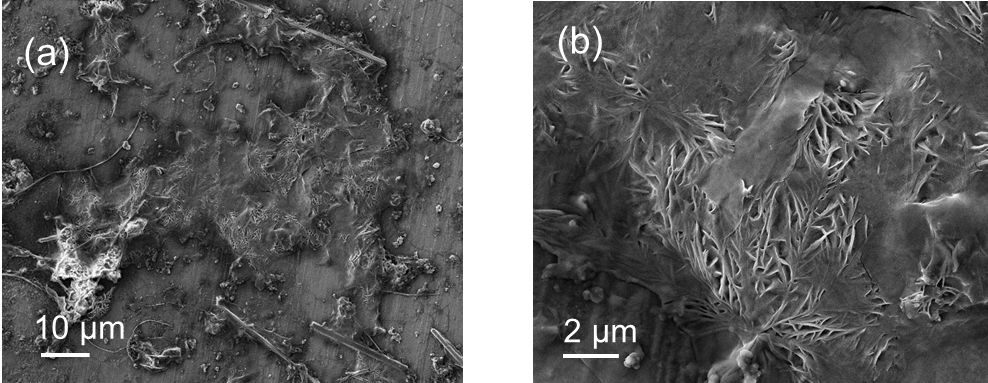
Figure S61** SEM images with different magnification of Zn plating/stripping at a current density of 1 mA cm^−2^ on 20 h.

**
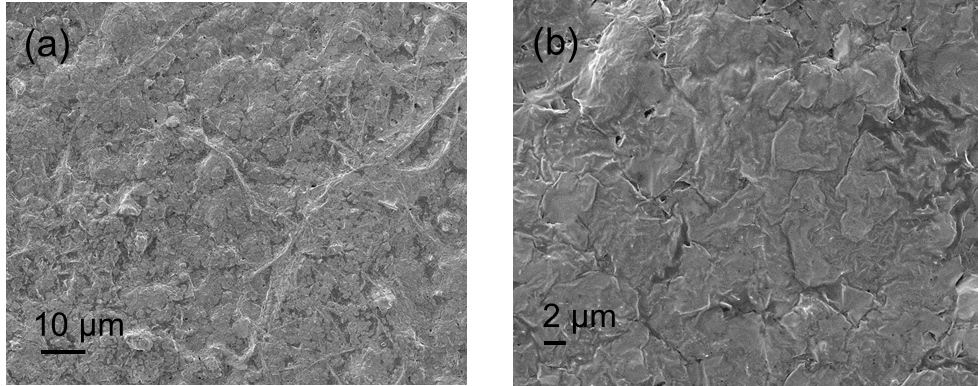
**

**Figure S****62** SEM images with different magnification of Sn@BIBCs-Zn plating/stripping at a current density of 5 mA cm^−2^ at 100 h.

**
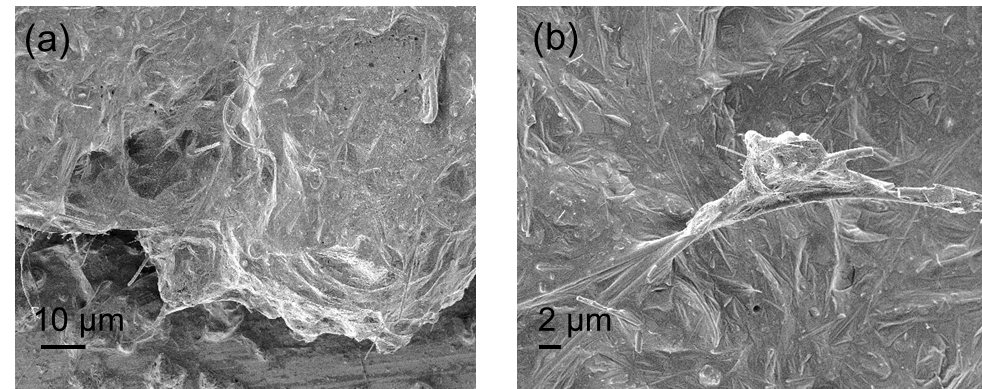
**

**Figure S63** SEM images with different magnification of Zn plating/stripping at a current density of 5 mA cm^−2^ at 100 h.

**
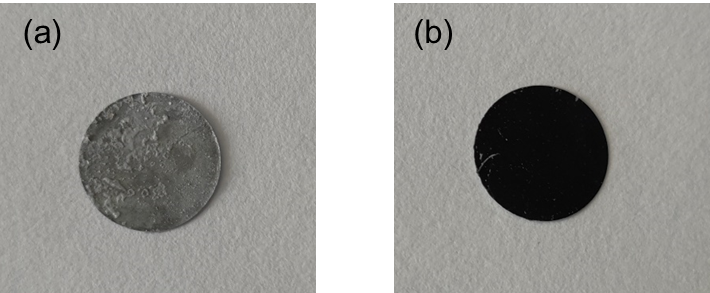
**

**Figure S64** Optical photo images of (a) bare Zn foil and (b) Sn@BIBCs-Zn after immersion in the aqueous ZnSO_4_ electrolyte for 72 h.

**
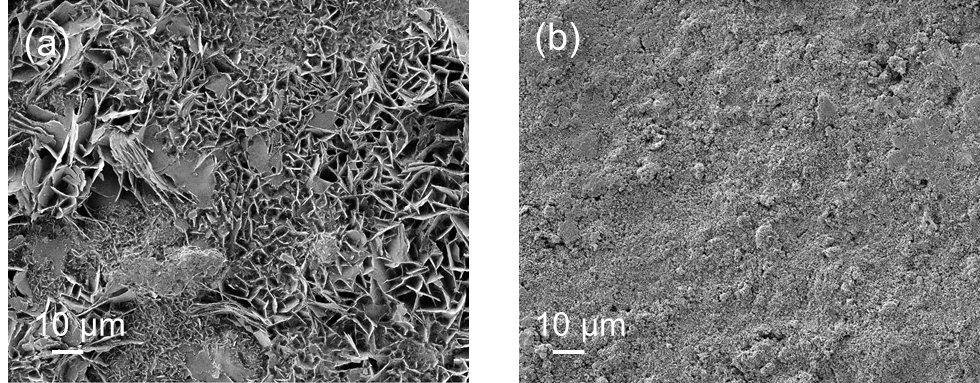
**

**Figure S65** SEM images of (a) bare Zn foil and (b) Sn@BIBCs-Zn after immersion in the aqueous ZnSO_4_ electrolyte for 72 h.

**1.10 Supplementary electrochemical performance of Zn-Se full batteries.**


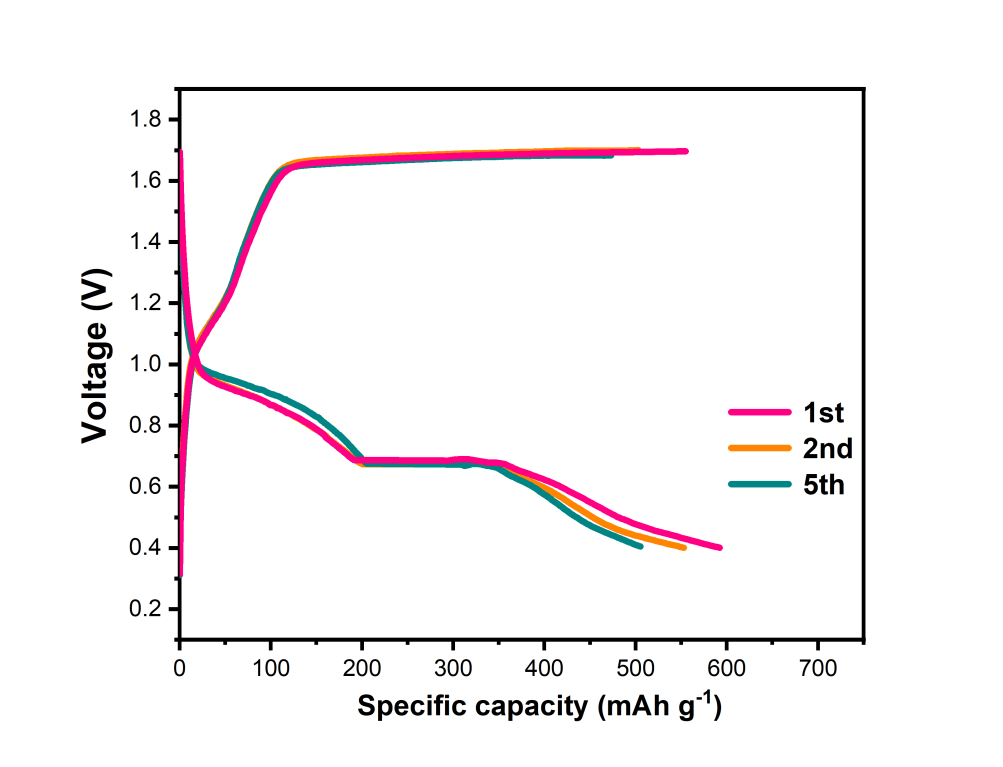


**Figure S66** GCD profiles at 0.1 C of the Sn@BIBCs-Zn||Se-SnSe@BIBCs cell.

Note: As shown in Figure S66, the discharge plateau at 0.7 V corresponds to the reduction reaction of Se^0^ to Se^2-^, while the charging plateau at 1.7 V corresponds to the oxidation reaction of Se^0^ to Se^4+^.

**Table S1** Specific surface area of all samples

|  |  |  |  |
| --- | --- | --- | --- |
| Materials | DWHCSs | BIBCs | SnO_2_@BIBCs |
| Specific surface area (m^2^ g^−1^) | 789.2 | 1015.4 | 641.1 |
| Materials | Sn@BIBCs | SnSe@BIBCs | Se-SnSe@BIBCs |
| Specific surface area (m^2^ g^−1^) | 417.7 | 580.5 | 344.7 |

**Table S2** Charge transfer rate of all samples

| Materials | SnSe | graphene | MoSe_2_ | SnS_2_ | SnSe_2_ |
| --- | --- | --- | --- | --- | --- |
| Charge transfer rate (eV) | 0.422 | 0.226 | 0.388 | 0.238 | 0.267 |

**Table S3** Nucleation overpotential of all samples

| Materials | DWHCSs | BIBCs | HCSs |
| --- | --- | --- | --- |
| nucleation overpotential (mV) | 66.1 | 49.6 | 68.9 |
| Materials | Sn@BIBCs | Sn@DWHCSs | Ti foil |
| nucleation overpotential (mV) | 20.5 | 38.5 | 78.3 |
